# Targeting TTK Inhibits Tumorigenesis of T‐Cell Lymphoma Through Dephosphorylating p38α and Activating AMPK/mTOR Pathway

**DOI:** 10.1002/advs.202413990

**Published:** 2025-01-21

**Authors:** Bingyu Liu, Tiange Lu, Mengfei Ding, Xiaoli Zhou, Yujie Jiang, Juanjuan Shang, Wenyue Sun, Shunfeng Hu, Xin Wang, Xiangxiang Zhou

**Affiliations:** ^1^ Department of Hematology Shandong Provincial Hospital Cheeloo College of Medicine Shandong University Jinan Shandong 250021 China; ^2^ Department of Hematology Shandong Provincial Hospital Affiliated to Shandong First Medical University Jinan Shandong 250021 China; ^3^ Branch of National Clinical Research Center for Hematologic Diseases Jinan Shandong 250021 China

**Keywords:** p38α, phosphorylation, T‐cell lymphoma, threonine tyrosine kinase, ubiquitination

## Abstract

T‐cell lymphoma (TCL) is a group of non‐Hodgkin's lymphoma with high heterogeneity and unfavorable prognosis. Current standard treatments have demonstrated limited efficacy in improving the outcomes for TCL patients. Therefore, identification of novel drug targets is urgently needed to improve the prognosis of TCL patients. Through multi‐omics analysis, aberrant expression of threonine tyrosine kinase (TTK) in TCL is identified. High expression of TTK is closely associated with poor prognosis in TCL patients. Targeting TTK through gene knockdown exerts anti‐tumor effects in vitro and in vivo, including inhibiting the cell proliferation, inducing G2/M phase arrest, enhancing DNA damage and cell apoptosis. Mechanically, p38α is identified as the potential phosphorylation substrate of TTK through phosphoproteomic quantification and motif prediction. Furthermore, inhibition of TTK suppresses activation of p38α through dephosphorylating it at Thr180/Tyr182, thereby promoting the activation of AMPK/mTOR pathway. In addition, targeting TTK enhances the autophagy in TCL cells through dephosphorylating p38α. CFI‐402257, a specific inhibitor of TTK, is found to exhibit anti‐tumor effects and exerted synergistic efficacy with PI3K inhibitor, Duvelisib, in TCL. The study shows that TTK contributes to the development of TCL by regulating p38α‐mediated AMPK/mTOR pathway. CFI‐402257 is expected to be a promising strategy for TCL treatment.

## Graphical abstract

In the study, aberrant expression of threonine tyrosine kinase (TTK) in TCL is identified. High expression of TTK is closely associated with poor prognosis in TCL patients. Inhibition of TTK, by either knockdown or specific inhibitor CFI‐402257, exerts anti‐tumor effects in vitro and in vivo. Mechanically, targeting TTK induces autophagy through dephosphorylating p38α at Thr180/Tyr182, which further promotes the activation of AMPK/mTOR pathway.

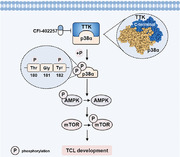

## 1 Introduction

T‐cell lymphoma (TCL) is a group of malignancies that arises from T cells, which is characterized by high aggressiveness and poor prognosis.^[^ ^1^ ^]^ The complex molecular subtyping has resulted in the absence of specific and highly effective targeted therapy for TCL patients. Despite the development of new drugs including CD30 antibody‐drug conjugate, histone deacetylase inhibitors (HDACi), and folic acid antagonists,^[^ ^2^ ^]^ the therapeutic outcome and prognosis of TCL patients remain unsatisfactory with 5‐year progression‐free survival (PFS) rate less than 40% and overall survival (OS) rate less than 50% in peripheral T‐cell lymphoma (PTCL), while the 5‐year OS of T‐cell acute lymphoblastic leukemia/lymphoma (T‐ALL) was 48%.^[^ ^3^ ^]^ Given the lack of effective therapeutic interventions, advancing our understanding of the molecular biology in TCL and identifying novel drug targets are urgently needed to optimize the clinical outcome of TCL patients.

Genomic instability is a hallmark of tumor cells, contributing to aberrant cell cycle control and indeterminate proliferation through the dysregulation of essential cellular pathways.^[^ ^4^ ^]^ The spindle assembly checkpoint (SAC) has been reported as a critical biological function to ensure accurate chromosome segregation in mitosis and prevent genomic instability.^[^ ^5^ ^]^ Targeting SAC proteins could induce high levels of genomic instability and aneuploidy that tumor cells cannot tolerate, ultimately leading to senescence and apoptosis.^[^ ^6^ ^]^

Threonine tyrosine kinase (TTK) is the central molecule that plays a crucial role in recruiting other SAC proteins to unattached kinetochores in prometaphase.^[^ ^6^ ^]^ More importantly, TTK has been found to regulate the phosphorylation of various substrates including c‐Abl,^[^ ^7^ ^]^ MDM2,^[^ ^8^ ^]^ and CHK2^[^ ^9^ ^]^ through kinase activity, thereby mediating cellular oxidative stress and DNA damage repair. Upregulation of TTK has been observed in various tumors, such as breast cancer and hepatocellular carcinoma (HCC), and was associated with worse prognosis of tumor patients.^[^ ^6^, ^10^ ^]^ Moreover, TTK specific inhibitors, including NMS‐P715, MPI‐0479605, MPS‐BAY2b, and MPS‐IN‐3, have been found to exert anti‐tumor effects in various tumors.^[^ ^11^, ^12^, ^13^ ^]^ CFI‐402257 is a novel developed TTK inhibitor with higher selectivity,^[^ ^14^ ^]^ which has obtained approval for a phase 2 clinical trial involving patients with ER+/HER2‐ advanced breast cancer (NCT05251714).^[^ ^15^, ^16^ ^]^ However, the roles and mechanisms of TTK and its inhibitors in hematologic malignancies, particularly TCL, have not been clarified and require further investigation.

In this study, we identified TTK as a pivotal contributor to the development of TCL. Targeted inhibition of TTK exerted anti‐tumor effects both in vitro and in vivo. Mechanically, inhibiting TTK dephosphorylated p38α at Thr180/Tyr182, thereby promoting the activation of AMPK/mTOR pathway. TTK inhibitor, CFI‐402257, exerted anti‐tumor effects in TCL and synergized with the PI3K inhibitor Duvelisib. These results elucidated the oncogenic role of TTK in TCL and provided a novel potent target and combinatorial therapeutic strategy for TCL patients.

## 2 Results

### 2.1 TTK Expression Was Upregulated in TCL and Associated with Tumor Progression

To screen for differentially expressed genes (DEGs) in TCL, analysis using multi‐omics data from publicly available databases was conducted. Through comparing 142 TCL clinical samples to 26 normal T cell samples from healthy donors, DEGs analysis revealed significant upregulation of 3967 genes and downregulation of 5969 genes in TCL (Fold Change > 1.2, *P* < 0.05, Figure S1A,B, Supporting Information). Furthermore, Kyoto Encyclopedia of Genomes (KEGG) pathway enrichment analysis demonstrated significant enrichment of DEGs in tumor‐associated signaling pathways including PI3K/AKT pathway and mTOR pathway (Figure S1C, Supporting Information). Kaplan–Meier survival analysis of 192 TCL patients identified 2040 prognostic‐associated genes in TCL (*P* < 0.05, Figure S1D, Supporting Information). Moreover, 1203 survival‐critical genes in four TCL cell lines (Karpas‐299, Ki‐JK, SUP‐M2, SUP‐T1) were identified using CRISPR screen data (Figure S1E, Supporting Information). Combined with the above results, we disclosed 13 genes that might play key roles in TCL progression, including TTK, CDCA8, BUB1B, HJURP, HIST1H2BM, TICAM2, KIF23, CDT1, RPP40, CRCP, SDC1, HAMP, RAD51 (Figure S1F, Supporting Information).

**Figure 1:**
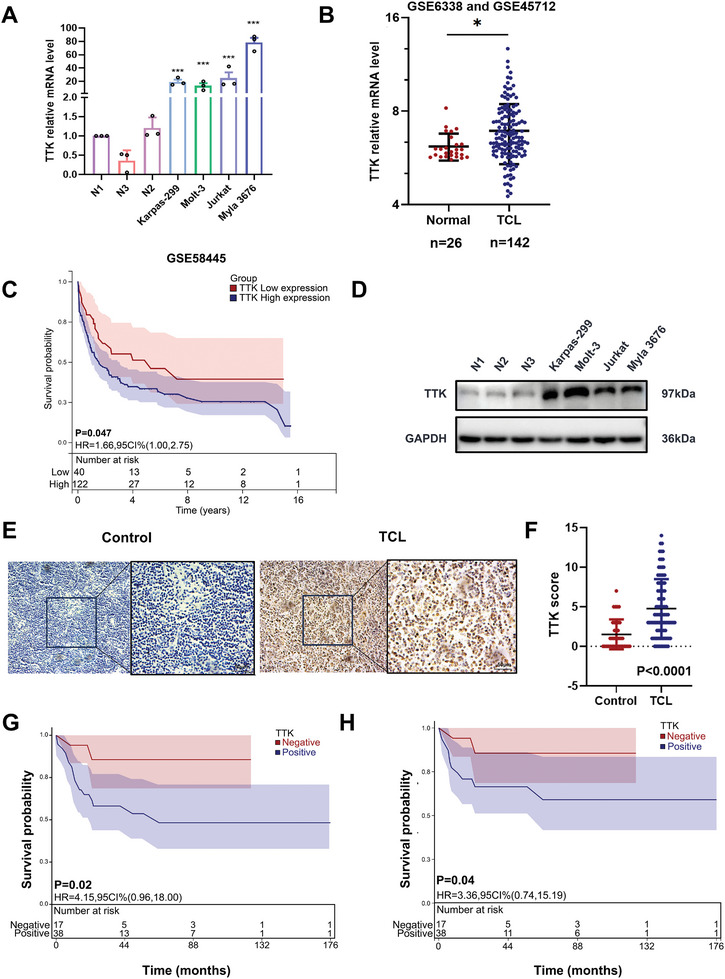
TTK expression was upregulated in TCL and associated with tumor progression. A) qPCR analysis showed that TTK expression was higher in TCL cells compared with normal T cells from healthy donors (n = 3). The level of TTK mRNA is shown on the y‐axis, while the cells are displayed on the x‐axis. B) The levels of TTK mRNA were significantly increased in TCL tissues (n =  142) from GSE6338 and GSE45712 compared with normal T cells (n  = 26). C) Kaplan–Meier analysis showed that high TTK expression level was associated with poor OS in TCL patients (n = 192) from GSE58445. D) WB showed that the expression level of TTK was higher in TCL cell lines compared with T cells from healthy donors. E) Representative IHC staining images of TTK in TCL tissues and reactive hyperplasia samples. Bar  =  50 µm. F) TTK expression was higher in TCL tissues (n  =  92) compared to reactive hyperplasia samples (n  =  40, Control) when detected by IHC. G‐H) Kaplan–Meier survival analysis showed the association between TTK expression and OS and PFS in TCL patients, respectively. Data are shown as the mean ± SD. *P <  0.05; **P <  0.01; ***P <  0.001.

Interestingly, cell cycle has been enriched in the gene function enrichment of DEGs, prognostic‐associated genes and survival‐critical genes (Figure S2A–C, Supporting Information), suggesting that cell cycle regulation may be closely associated with the development of TCL. To further investigate the effect of cell cycle regulation in TCL development, drug screening was performed based on FDA Anti‐tumor Drug Library and 110 drugs were identified to effectively inhibit TCL cell viability. Among them, drugs targeting cell cycle, cytoskeletal signaling, and NF‐κB showed the highest effective ratio (Figure S2D, Supporting Information). Consequently, we specifically focused on 4 cell cycle‐related genes, including TTK, BUB1B, CDT1, RAD51, and further performed qPCR to verify their expression levels in TCL (Figure S2E, Supporting Information; **Figure** **1**A). Consequently, TTK mRNA was consistently upregulated in TCL compared to peripheral blood normal T cells from healthy donors and displayed the highest expression level among the four genes (Figure 1A).

Given the aberrant expression of TTK, we further explored the potential value of TTK in TCL. Up‐regulated TTK mRNA level was found in TCL patients (Figure 1B). Survival analysis indicated that TTK mRNA expression was associated with poor prognosis (Figure 1C). Significantly higher protein level of TTK was observed in TCL cell lines, compared to normal T cells from healthy donors (Figure 1D). The elevated TTK protein expression was verified by immunohistochemical (IHC) staining in TCL primary tissues (n = 92) compared to reactive hyperplasia (n = 40) (Figure 1E,F).

Further survival analysis revealed the correlation between high TTK expression and worse OS and PFS in Shandong Provincial Hospital cohort (Figure 1G,H). The relevance between TTK expression and clinicopathological parameters of TCL patients was evaluated and TTK positive expression was associated with elevated plasma salivary acid (Table S1, Supporting Information). Collectively, above results suggested that the expression of TTK might serve as a promising prognostic factor in TCL patients.

### 2.2 Knockdown of TTK Suppressed the Growth of TCL In Vitro and In Vivo Through Regulating Cell Cycle, DNA Damage and Apoptosis

**Figure 2:**
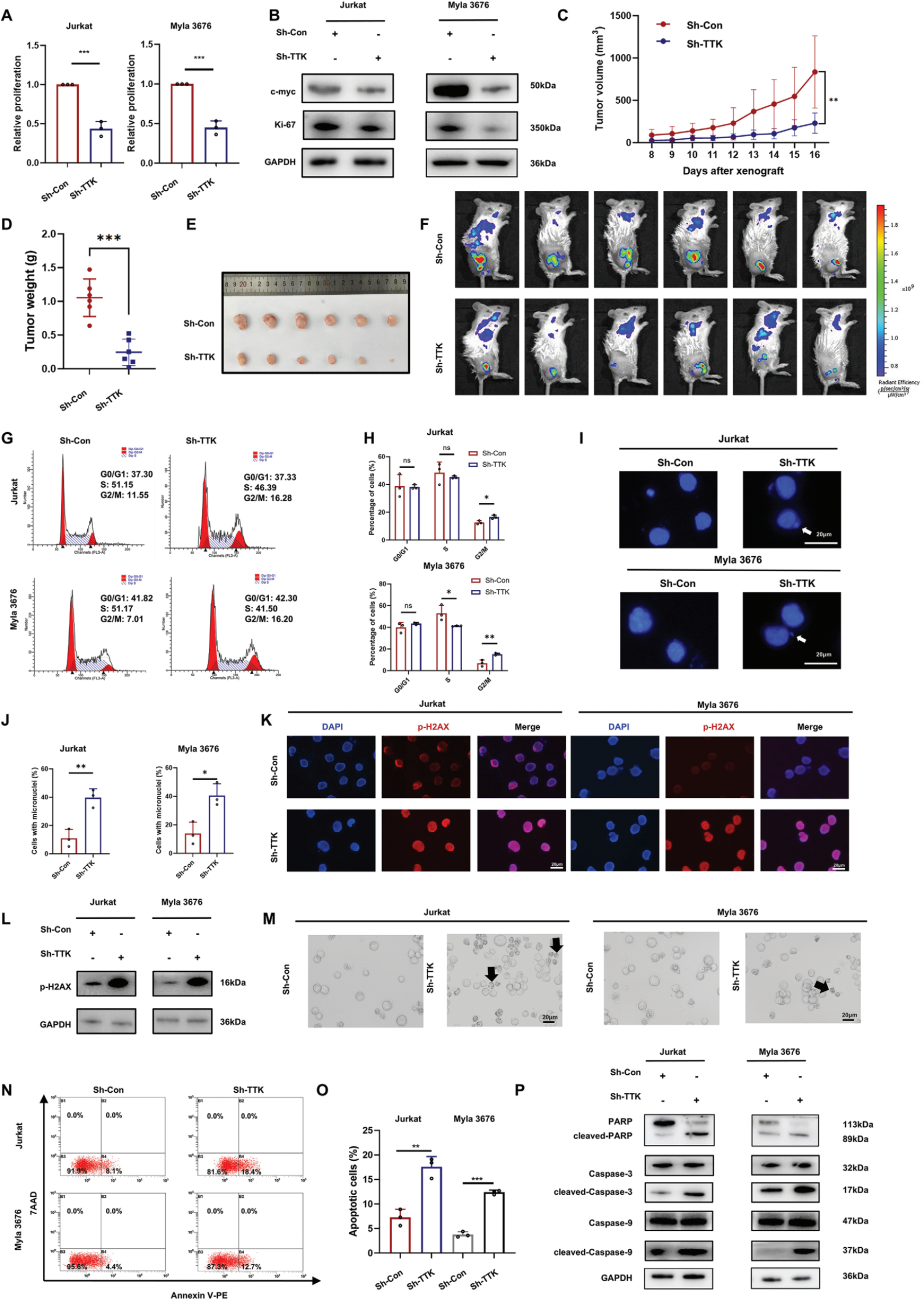
Knockdown of TTK suppressed the growth of TCL in vitro and in vivo through regulating cell cycle, DNA damage and apoptosis. A) TTK knockdown inhibited the cell proliferation of TCL cells when assessed using CCK8 assay (n = 3). B) WB assay was used to detect the expression level of c‐myc and Ki‐67. TTK knockdown inhibited c‐myc and Ki‐67 expression in TCL cells. C) Time course of tumor growth in Sh‐Control and Sh‐TTK TCL xenograft models (n  =  6 per group). D) Tumor weights of xenografts taken from TCL xenograft models in Sh‐Control and Sh‐TTK groups (n  =  6 per group). E) Tumor volumes of xenografts taken from TCL mouse models in Sh‐Control and Sh‐TTK groups (n  =  6 per group). F) Bioluminescence of tumor in Sh‐Control and Sh‐TTK TCL xenograft models (n  =  6 per group). G‐H) Detection of cell cycle in TCL cells after TTK knockdown by flow cytometry (n = 3). I‐J) Immunofluorescence analysis showed that TTK knockdown increased the number of micronuclei in TCL cells. Bar  =  20 µm. K) Immunofluorescence analysis showed that TTK knockdown enhanced the intensity of p‐H2AX in TCL cells. Bar  =  20 µm. L) WB analysis of the expression level of p‐H2AX in TCL cells after TTK knockdown. M) Representative images of cell death after TTK knockdown in TCL cells. Bar  =  20 µm. N‐O) Detection of cell apoptosis in TCL cells after TTK knockdown by flow cytometry (n = 3). P) WB analysis of the expression level of apoptotic markers in TCL cells after TTK knockdown. Data are shown as the mean ± SD. **P < 0.05; **P < 0.01; ***P < 0.001.

The above findings motivated us to further explore the biological function of TTK in TCL. TTK knockdown and overexpression models were established in both Jurkat and Myla 3676 cells (Figure S3A–C, Supporting Information). Knockdown of TTK significantly inhibited the cell proliferation of TCL cells (**Figure** **2**A). The expression of proliferation marker, c‐myc and Ki‐67, were also found to be inhibited after TTK knockdown (Figure 2B). To further confirm the regulatory role of TTK on TCL progression in vivo, we constructed TCL xenograft mouse models (n = 6 per group). Compared with the control group, mouse bearing Sh‐TTK cells displayed reduced growth rate of tumor (Figure 2C), tumor weight (Figure 2D), and tumor volume (Figure 2E). Besides, mouse receiving Sh‐TTK TCL cells displayed decreased level of tumor burden when estimated by in vivo imaging system (Figure 2F). IHC results showed that Sh‐TTK group displayed reduced Ki‐67 expression level (Figure S3D, Supporting Information). Taken together, our findings indicated that TTK contributed to TCL growth both in vitro and in vivo.

To further explore the potential effects of TTK in TCL, functional enrichment analysis of DEGs between TCL patients with high and low TTK expression was performed in GSE6338 and GSE45712. Gene ontology (GO) analysis revealed that TTK appeared to be closely related to cell proliferation, apoptosis, cell cycle, chromosome segregation and DNA damage repair (Figure S3E, Supporting Information). To verify the biological role of TTK in TCL, flow cytometry was employed and the results revealed that TTK knockdown led to the accumulation of cells in G2/M phase in TCL cells (Figure 2G,H). Given the crucial role of TTK in normal chromosome segregation during mitosis, immunofluorescence (IF) staining of α‐tubulin was performed and showed enhanced chromosome missegregation in TCL cells after TTK knockdown (Figure S3F, Supporting Information). As micronuclei, chromosome fragments encased by the nuclear membrane in the cytoplasm, is usually acknowledged to arise from mitotic defects, we also found that TTK knockdown promoted the formation of micronuclei in TCL (Figure 2I,J), which may be mediated by chromosome missegregation and resulted in enhanced DNA damage. Furthermore, TTK knockdown also increased the expression level of p‐H2AX, a marker of DNA damage, in TCL (Figure 2K,L).

It has been reported that DNA damage could induce p53‐dependent and independent apoptosis,^[^ ^17^ ^]^ the effect of TTK knockdown on apoptosis in TCL cells was further investigated. Microscopic examination found that TTK knockdown caused cell death manifested by the formation of solidified chromatin and cleaved nuclei (Figure 2M). Flow cytometry also revealed a significant increase in the apoptotic ratio of TCL cells after TTK knockdown (Figure 2N,O). Additionally, TCL cells with TTK knockdown displayed increased expression of apoptosis‐related molecules, including cleaved‐PARP, cleaved‐Caspase‐3 and cleaved‐Caspase‐9, indicating the pro‐apoptosis role of TTK knockdown (Figure 2P). Above results suggested that targeting TTK might suppress the growth of TCL through inducing cell cycle arrest, DNA damage and apoptosis.

### 2.3 TTK Specific Inhibitor CFI‐402257 Displayed Anti‐Tumor Effects in TCL

**Figure 3:**
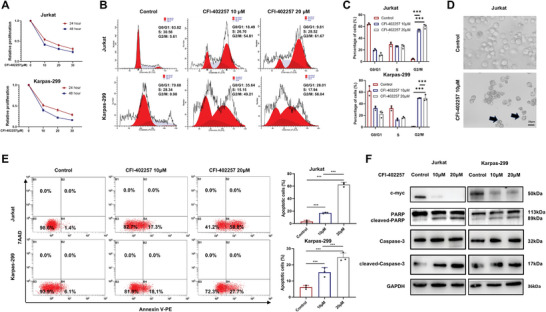
TTK specific inhibitor CFI‐402257 displayed anti‐tumor effects in TCL. A) CCK8 assay showed that CFI‐402257 exerted time‐ and dose‐dependent inhibitory effect on the proliferation in TCL cells (n = 3). B‐C) Detection of cell cycle in TCL cells after CFI‐402257 treatment (10 µM or 20 µM, 48 h) by flow cytometry (n = 3). D) Representative images of cell death after CFI‐402257 treatment (10 µM, 48 h) in TCL cells. Bar  =  20 µm. E) Detection of cell apoptosis in TCL cells after CFI‐402257 treatment (10 µM or 20 µM, 48 h) by flow cytometry (n = 3). F) WB analysis showed that CFI‐402257 treatment (10 µM or 20 µM, 48 h) decreased the expression of c‐myc and increased the expression of apoptotic markers in TCL cells. Data are shown as the mean ± SD. *P <  0.05; **P <  0.01; ***P <  0.001.

To further investigate the potential anti‐tumor effects of targeting TTK in TCL, we used CFI‐402257 to selectively inhibit the activity of TTK. Time‐ and dose‐dependent inhibitory effect of CFI‐402257 treatment on the proliferation was found in TCL cells (**Figure** **3**A). The inhibitory effect of CFI‐402257 on cell viability was also observed in TCL cells (Figure S4A, Supporting Information). Besides, CFI‐402257 was found to induce the G2/M phase arrest in TCL cells (Figure 3B,C). Moreover, CFI‐402257 treatment resulted in abnormal chromosome segregation in TCL cells (Figure S4B, Supporting Information). Elevated micronuclei formation and p‐H2AX expression were also observed through IF staining and WB (Figure S4C–E, Supporting Information). CFI‐402257 induced cell death manifested by solidified chromatin and cleaved nuclei (Figure 3D). In addition, flow cytometry results revealed increased rate of apoptotic TCL cells after CFI‐402257 treatment (Figure 3E). Furthermore, downregulation of c‐myc expression and upregulation of cleaved‐PARP, cleaved‐Caspase‐3 expression supported the anti‐proliferation and apoptosis‐inducing effects of CFI‐402257 in TCL cells (Figure 3F).

### 2.4 TTK Interacted with p38α Through C‐Terminal and Phosphorylated p38α in TCL

**Figure 4:**
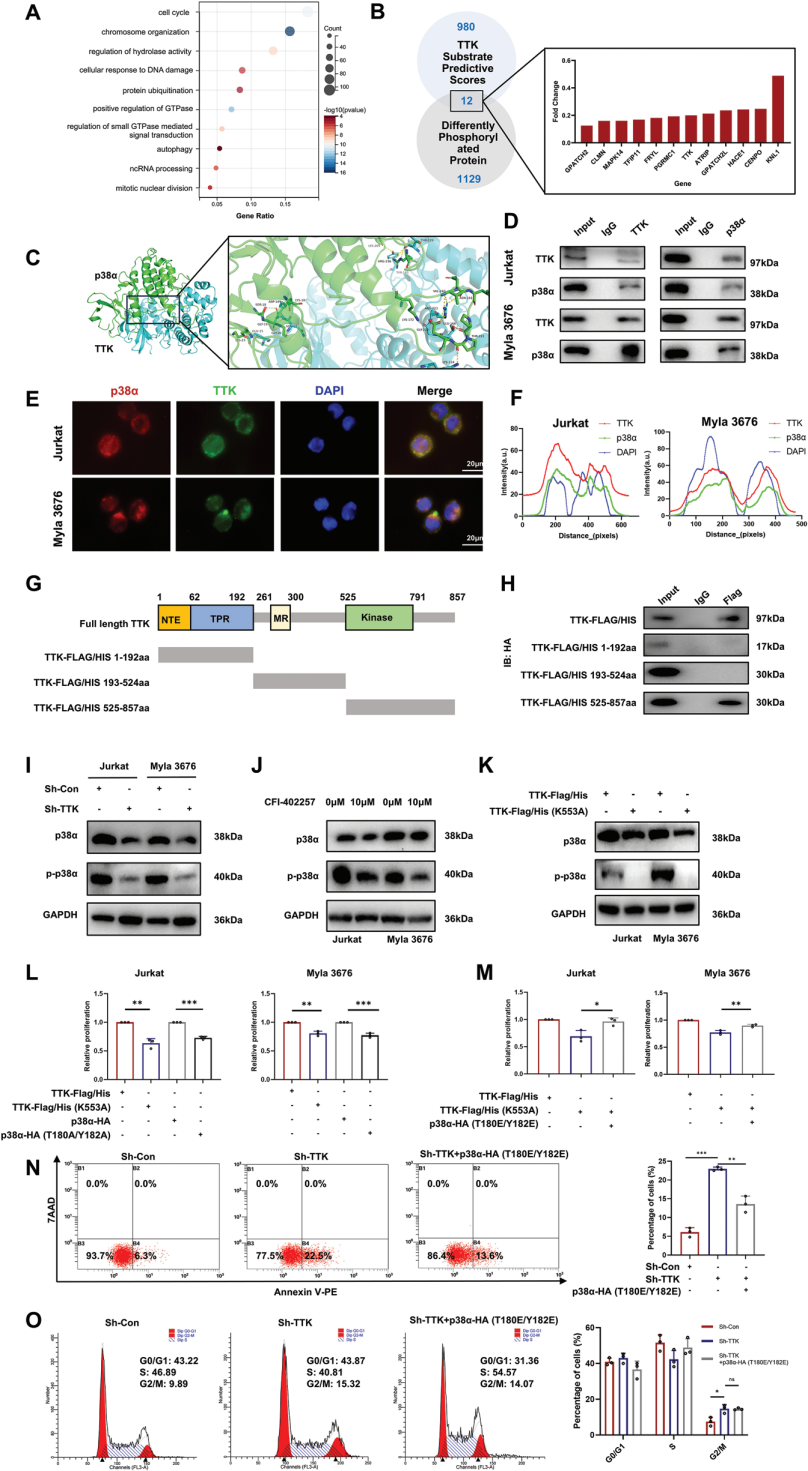
TTK interacted with p38α through C‐terminal and phosphorylated p38α in TCL. A) GO enrichment analysis of differentially phosphorylated proteins. The circle area indicated the number of genes, while the circle color represented the range of the corrected P values. B) 12 potential kinase substrates of TTK were identified by integrating the results of motif prediction percentile and phosphoproteomics quantification. Bar plot showed the fold change of differentially phosphorylated proteins. C) The predicted binding mode of TTK (blue) and p38α (green). D) Co‐IP assay showed the interaction between TTK and p38α in TCL. E) Immunofluorescence co‐staining of TTK and p38α in TCL. Bar  =  20 µm. F) Immunofluorescence distribution showed the co‐localization of TTK (red) and p38α (green). G) The schematic of TTK truncated fragments. H) Co‐IP assay confirmed the interaction between TTK 525–857aa and p38α in TCL. I) WB analysis showed the expression level of p38α and p‐p38α after TTK knockdown in TCL. J) WB analysis showed the expression level of p38α and p‐p38α after CFI‐402257 treatment (10 µM, 48 h) in TCL. K) WB analysis showed the expression level of p38α and p‐p38α after transfected with TTK kinase inactivated mutation plasmid. L‐M) CCK8 assay showed that the proliferation of TCL cells was inhibited by TTK kinase inactivation and p38 phosphorylation inactivation plasmids, which was reversed by p38 phosphorylation activation plasmid (n = 3). N) Detection of cell apoptosis in TCL cells after TTK knockdown and p38α phosphorylation activation plasmid transfection by flow cytometry (n = 3). O) Detection of cell cycle in TCL cells after TTK knockdown and p38α phosphorylation activation plasmid transfection by flow cytometry (n = 3). Data are shown as the mean ± SD. *P <  0.05; **P <  0.01; ***P <  0.001.

Previous studies have shown that TTK could regulate the progression of esophageal squamous cell carcinoma and melanoma through its kinase activity.^[^ ^18^, ^19^ ^]^ To delve deeper into the kinase regulatory mechanism of TTK in TCL, we conducted the quantification of the phosphoproteome in Jurkat cell and identified 1129 differentially phosphorylated proteins after TTK knockdown (Figure S5A,B, Supporting Information). These proteins were enriched in GO functions related to cell cycle, protein ubiquitination, autophagy, and so on (**Figure** **4**A). Additionally, to pinpoint the direct kinase substrates regulated by TTK, we integrated the results of motif prediction^[^ ^20^ ^]^ for TTK substrates and phosphoproteomics quantification, and identified 12 potential kinase substrates of TTK (Figure 4B).

Subsequently, to explore the possible interactions between TTK and potential substrates, HDOCK molecular docking analysis was performed and the Confidence Score of TTK and p38α was the highest (Figure 4C, Confidence Score = 0.9955, Docking score = ‐419.88, Ligand rmsd = 0.33). Co‐Immunoprecipitation (Co‐IP) further validated the protein‐protein interaction between TTK and p38α (Figure 4D). The fluorescence distributions in IF co‐staining of TTK and p38α supported their co‐localization within TCL cells (Figure 4E,F). Moreover, based on the reported TTK domains and their functions, we constructed three TTK truncated plasmids, N‐terminal‐K192 (including N‐terminal extension and N‐terminal tetratricopeptide repeat domains, associated with the subcellular localization of TTK to the centrosomes), Q193‐I524 (including MR domain, associated with the binding to NDC80 complex), and Y525‐C‐terminal (including kinase domain).^[^ ^21^, ^22^, ^23^ ^]^ Subsequently, full‐length and truncated plasmids of TTK were separately transfected in TCL cells companied by p38α‐HA plasmid (Figure 4G). It was disclosed by Co‐IP results that TTK interacted with p38α based on the C‐terminal region (Y525‐C‐terminal) (Figure 4H). These results suggested the interaction between TTK and p38α and their structural basis in TCL.

To further explore the regulatory mechanism between TTK and p38α, we assessed the relative expression level of p‐p38α after TTK knockdown, and found decreased expression of both p38α and p‐p38α in TCL cells, and the expression of p‐p38α was decreased with more prominent tendency (Figure 4I). Additionally, CFI‐402257 treatment was also found to decrease the expression of p38α and p‐p38α with the similar tendency (Figure 4J). Above results indicated that TTK had regulatory effect on both expression and phosphorylation of p38α in TCL. Given that TTK was initially identified as bispecific protein kinase phosphorylating tyrosine and threonine, to elucidate whether TTK regulated the expression of p38α through kinase activity, the phosphorylation inactivated (p38α‐HA, T180A/Y182A) and activated mutant (p38α‐HA, T180E/Y182E) plasmid of p38α and the kinase inactivated mutant plasmid of TTK (TTK‐Flag/His, K553A) were constructed. It was found that the transfection of TTK kinase inactivated mutant plasmid led to the downregulation of both p38α and p‐p38α expression in TCL, indicating that TTK regulated the expression and phosphorylation of p38α through kinase activity (Figure 4K). Collectively, the above results suggested that targeting TTK could inhibit p38α phosphorylation at T180/Y182.

Further rescue experiments were performed to explore whether the regulatory role of TTK on TCL proliferation depended on the phosphorylation of p38α. The proliferation of TCL cells was inhibited by the transfection of TTK kinase inactivation and p38 phosphorylation inactivation plasmids, whereas the p38α phosphorylation activation plasmid reversed the inhibitory effect of TTK kinase inactivation plasmid on proliferation (Figure 4L,M), suggesting that TTK exerted pro‐proliferative effects in TCL through the phosphorylation of p38α. Moreover, we also found that p38α phosphorylation activation plasmid reversed the pro‐apoptotic effect of TTK knockdown in TCL when detected by flow cytometry (Figure 4N). However, p38α phosphorylation activation failed to reverse the G2/M phase arrest after TTK knockdown, indicating that TTK regulated the cell cycle mainly in a p38α‐independent manner (Figure 4O).

Notably, as TTK inhibition and kinase inactivated mutation also decreased the level of p38α, to further explore the regulatory mechanism of TTK on p38α expression, qPCR was performed and TTK knockdown did not significantly change the mRNA expression level of MAPK14 (p38α) (Figure S6A, Supporting Information), indicating that TTK might directly regulate the protein level of p38α. To explore whether TTK affected the protein degradation of p38α, we conducted Cycloheximide (CHX) assay and illustrated that the protein degradation of p38α was expedited after TTK knockdown, which could be reversed by MG132 (Figure S6B, Supporting Information). Given that p38α phosphorylation has been reported to regulate the activity of ubiquitin protein ligase including PJA1 and Ube3c,^[^ ^24^, ^25^ ^]^ it was hypothesized that the phosphorylation of p38α could affect its own ubiquitination degradation. We subsequently conducted CHX assay after transfecting WT p38α (p38α‐HA), phosphorylation‐inactivated (p38α‐HA, T180A/Y182A), and activated p38α plasmids (p38α‐HA, T180E/Y182E) to investigate the association between p38α phosphorylation and protein degradation. The results revealed that phosphorylation inactivation of p38α promoted the proteasome degradation of p38α while phosphorylation activation of p38α inhibited it (Figure S6C, Supporting Information). Additionally, Co‐IP analysis revealed that both TTK kinase inactivation and p38α phosphorylation inactivation promoted the ubiquitination of p38α in TCL (Figure S6D,E, Supporting Information). Subsequently, we predicted potential ubiquitination sites for p38α using GPS‐Uber^[^ ^26^ ^]^ and found that K152, K15, and K295 were the most promising sites for ubiquitination modifications (Table S2, Supporting Information). The above results implied that TTK regulated the ubiquitin‐proteasomal degradation of p38α via dephosphorylation‐coupled way.

### 2.5 Inhibiting TTK Activated the AMPK/mTOR Pathway Through p38α to Restrain TCL Development

**Figure 5:**
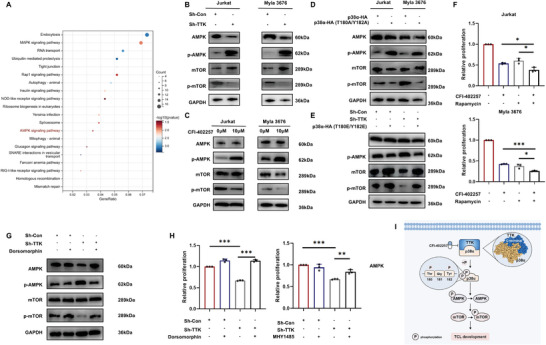
Inhibiting TTK activated the AMPK/mTOR pathway through p38α to restrain TCL development. A) KEGG enrichment analysis of differentially phosphorylated proteins. The circle area indicates the number of genes in the pathway, while the circle color represents the range of the corrected P values. B,C) WB analysis showed the expression level of p‐AMPK and p‐mTOR after TTK knockdown and CFI‐402257 treatment (10 µM, 48 h). D) WB analysis showed the expression level of p‐AMPK and p‐mTOR after transfection with p38α phosphorylation inactivation plasmid in TCL. E) WB analysis showed the expression level of p‐AMPK and p‐mTOR after TTK knockdown and transfection with p38α phosphorylation activation plasmid. F) CCK8 assay showed that the combined treatment with Rapamycin (mTOR inhibitor, 10 µM, 48 h) enhanced anti‐tumor effects of CFI‐402257 (10 µM, 48 h) in TCL (n = 3). G) WB analysis showed the expression level of p‐AMPK and p‐mTOR after TTK knockdown and Dorsomorphin (AMPK inhibitor) treatment (10 µM, 48 h). H) CCK8 assay showed that Dorsomorphin (AMPK inhibitor, 10 µM, 48 h) and MHY1485 (mTOR activator, 20 µM, 48 h) rescued the inhibitory effect of TTK knockdown on TCL cell proliferation (n = 3). I) Mechanism diagram summarized that TTK contributed to TCL development through regulating the p38α/AMPK/mTOR axis. Data are shown as the mean ± SD. *P <  0.05; **P <  0.01; ***P <  0.001.

To further pursue the downstream TTK‐regulated signaling pathway of TCL, KEGG enrichment analysis of differentially phosphorylated proteins after TTK knockdown revealed significant enrichment in the AMPK pathway (**Figure** **5**A). Subsequently, we found increased expression level of p‐AMPK and downregulation of p‐mTOR after inhibiting TTK, indicating the activation of AMPK/mTOR pathway (Figure 5B,C). Meanwhile, elevated expression levels of p‐TSC2 and p‐Raptor were also observed after TTK inhibition (Figure S7, Supporting Information). Furthermore, the expression level of p‐AMPK was also increased while p‐mTOR expression was decreased after transfection with p38α phosphorylation inactivation plasmid (Figure 5D). Importantly, the activation of AMPK/mTOR pathway after TTK knockdown could be rescued by the transfection with p38α phosphorylation activation plasmid (Figure 5E), indicating that TTK regulated the AMPK/mTOR pathway in TCL through phosphorylating p38α.

Additionally, considering the potential anti‐tumor effects of Rapamycin (mTOR inhibitor), we evaluated the combinational inhibitory effects of CFI‐402257 and Rapamycin on the proliferation of TCL cells, and found that the combined treatment with Rapamycin enhanced anti‐TCL effect of CFI‐402257 (Figure 5F). Furthermore, as TTK also regulated the expression of AKT, which could promote mTOR activation,^[^ ^27^, ^28^ ^]^ we used Dorsomorphin (AMPK inhibitor) to elucidate the main mechanism by which TTK regulated mTOR. The results displayed that Dorsomorphin treatment rescued the downregulation of p‐mTOR expression induced by TTK knockdown, suggesting that TTK mainly regulated mTOR in TCL via AMPK (Figure 5G). Subsequently, both Dorsomorphin and mTOR activator MHY1485 were found to rescue the inhibitory effect of TTK knockdown on TCL cell proliferation, suggesting that TTK modulated TCL proliferation through AMPK/mTOR pathway (Figure 5H). Collectively, above results suggested that targeting TTK activated the AMPK/mTOR pathway through p38α dephosphorylation to inhibit TCL development.

### 2.6 TTK Regulated the Autophagy in TCL via Modulating p38α Phosphorylation

**Figure 6:**
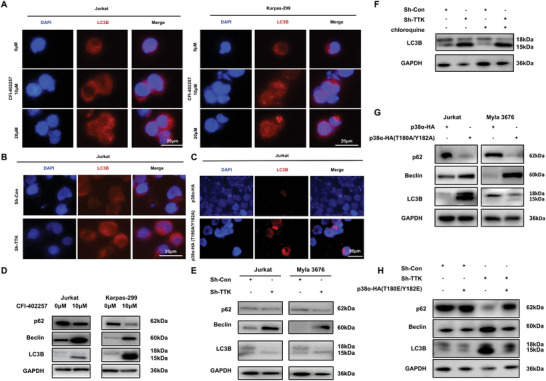
TTK regulated the autophagy in TCL via modulating p38α phosphorylation. A) IF analysis of LC3B autophagic vesicles (red) after CFI‐402257 treatment (10, 20µM, 48 h) in TCL cells. Bar  =  20 µm. B) IF analysis of LC3B autophagic vesicles (red) after TTK knockdown in TCL cells. Bar  =  20 µm. C) IF analysis of LC3B autophagic vesicles (red) after transfection with p38α phosphorylation inactivation plasmid in TCL. Bar  =  20 µm. D) WB analysis showed the expression level of p62, Beclin and LC3B‐II after CFI‐402257 treatment (10 µM, 48 h) in TCL cells. E) WB analysis showed the expression level of p62, Beclin, and LC3B‐II after TTK knockdown in TCL. F) WB was used to determine the expression level of p62, Beclin, and LC3B‐II after TTK knockdown and chloroquine treatment (10µM, 12 h) in TCL. G) WB analysis showed the expression level of p62, Beclin, and LC3B‐II after transfection with p38α phosphorylation inactivation plasmid in TCL. H) WB analysis showed the expression level of p62, Beclin and LC3B‐II after TTK knockdown and transfection with p38α phosphorylation activation plasmid in TCL. Data are shown as the mean ± SD. *P < 0.05; **P < 0.01; ***P < 0.001.

Differentially phosphorylated proteins after TTK knockdown were significantly enriched in biological processes including autophagy as observed in the functional enrichment. Meanwhile, it has been reported that p38α was involved in the autophagy regulation.^[^ ^29^, ^30^ ^]^ To elucidate whether TTK regulated autophagy in TCL cells through p38α, we detected the autophagic vesicles after TTK knockdown. IF staining revealed the augmentation of LC3B in the autophagic vesicles after CFI‐402257 treatment (**Figure** **6**A). LC3B IF staining also revealed that TTK knockdown and p38α phosphorylation inactivation plasmid transfection promoted autophagy process in TCL cells (Figure 6B,C; Figure S8, Supporting Information). CFI‐402257 treatment and TTK knockdown reduced the expression level of p62 and elevated the expression level of Beclin and LC3B‐II (Figure 6D,E). Chloroquine was used to block autophagosome degradation and it was found that chloroquine treatment further enhanced the promotion of autophagy by TTK knockdown, suggesting that TTK knockdown enhanced the autophagic flux in TCL (Figure 6F). Moreover, assessment of p62, Beclin, LC3B‐II also revealed enhanced autophagy process in TCL cells after p38α phosphorylation inactivation (Figure 6G). Notably, p38α phosphorylation activation plasmid rescued the effect of TTK knockdown on autophagy, indicating that TTK regulated autophagy by phosphorylating p38α in TCL (Figure 6H). Taken together, our results clarified that TTK could regulate the autophagy in TCL via modulating p38α phosphorylation.

### 2.7 CFI‐402257 Exerted Synergetic Anti‐Tumor Effects with Duvelisib in TCL Cells and Xenograft Models

**Figure 7:**
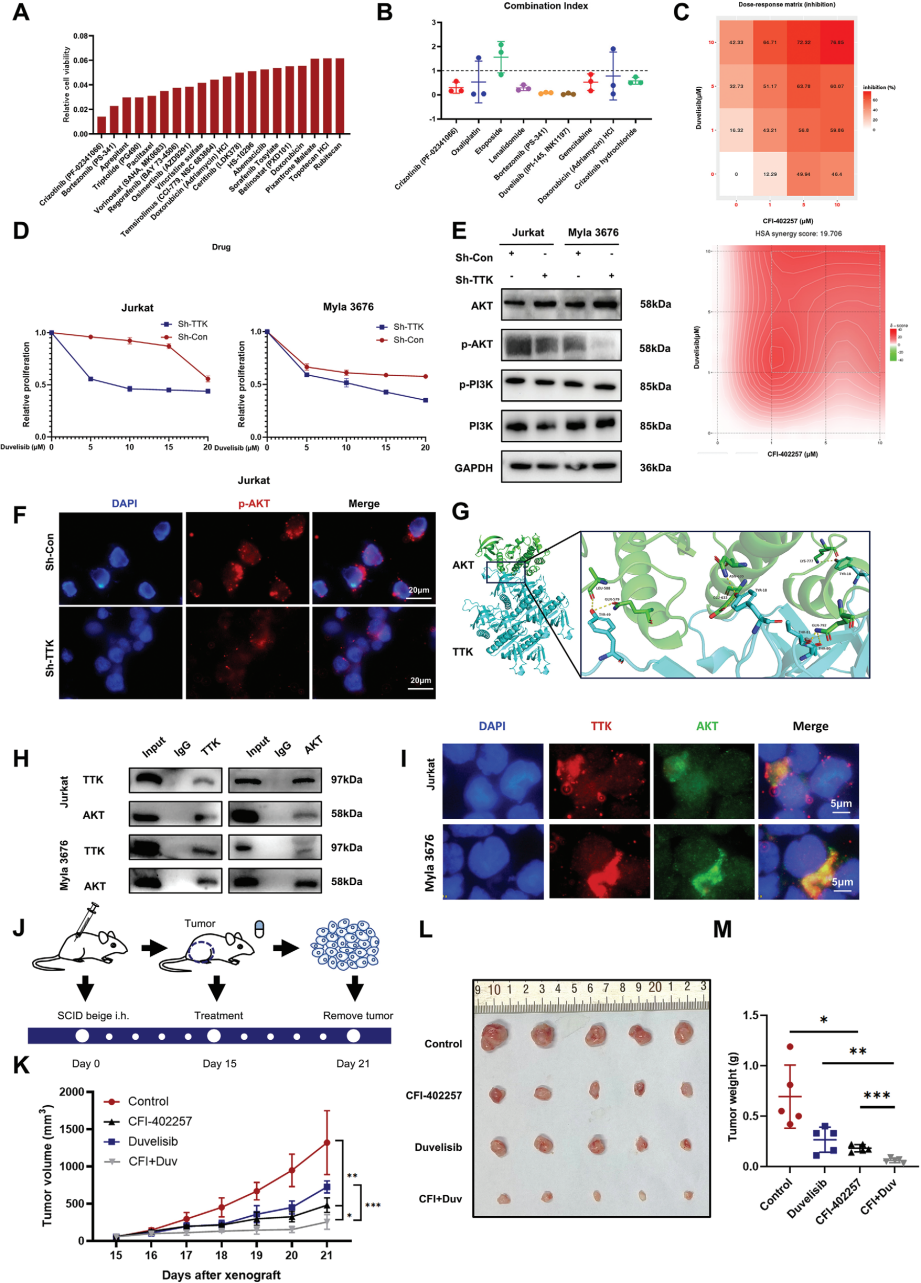
CFI‐402257 exerted synergetic anti‐tumor effects with Duvelisib in TCL cells and xenograft models. A) Combinational inhibitory effect of drugs in FDA Anti‐tumor Drug Library with 10 µM CFI‐402257 in Jurkat cell after 48 h treatment. Plot showed the top 20 drugs with the lowest relative cell viability. B) Chou–Talalay Combination Index of 9 drugs with 10 µM CFI‐402257 in Jurkat cell after 48 h treatment. C) HSA synergetic scores of Duvelisib with 10 µM CFI‐402257 in Jurkat cell after 48 h treatment. D) CCK8 assay showed that TTK knockdown increased the sensitivity of TCL cells to Duvelisib (n = 3). E) WB analysis showed the expression of p‐PI3K and p‐AKT after TTK knockdown in TCL. F) IF analysis of p‐AKT on cell membrane after TTK knockdown in TCL. G) The predicted binding mode of TTK (blue) and AKT (green). H) Co‐IP analysis showed the interaction between TTK and AKT in TCL cells. I) IF co‐staining of TTK and AKT in TCL. Bar  =  5 µm. J) Schematic diagram of the TCL xenograft mouse models treated with CFI‐402257 (6mg kg^−1^ day^−1^) and Duvelisib (10mg kg^−1^ day^−1^). K) Time course of tumor growth in TCL xenograft models treated with CFI‐402257 and Duvelisib (n  =  5 per group). L) Tumor volumes of xenografts taken from TCL mouse models treated with CFI‐402257 and Duvelisib (n  =  5 per group). M) Tumor weights of xenografts taken from TCL mouse models treated with CFI‐402257 and Duvelisib (n  =  5 per group). Data are shown as the mean ± SD. *P < 0.05; **P < 0.01; ***P < 0.001.

To further explore the potential clinical application of CFI‐402257 in TCL treatment, 211 drugs with anti‐tumor effects in TCL from FDA Anti‐tumor Drug Library were selected to uncover the potential synergetic drugs with CFI‐402257 according to single drug anti‐TCL effect and drug recommendation in clinical practice. The combinational inhibitory effect of 211 drugs with 10µM CFI‐402257 in TCL cell was evaluated by CellTiter‐Glo Luminescent (CTG) assay and the top20 drugs were shown in **Figure** **7**A. Subsequently, 9 TCL clinical recommended drugs with potential combination effects were used to assess the Chou‐Talalay Combination Index (CI) with CFI‐402257 at three concentration gradients (Figure 7B). Three drugs (Duvelisib, Lenalidomide, Bortezomib) with the smallest mean CI at all three concentration gradients were selected to further evaluate the synergetic effect with different combinational concentration ratios of CFI‐402257. It was found that Duvelisib (the PI3K δ/γ inhibitor) exhibited the highest synergetic scores, indicating its intense and stable synergetic effect with CFI‐402257 in TCL (Figure 7C; Figure S9A–C, Supporting Information). Moreover, TTK knockdown was found to enhance the sensitivity of TCL cells to Duvelisib, indicating that TTK was involved in the mechanism of synergistic effects (Figure 7D).

As Duvelisib is the PI3K δ/γ inhibitor, we further examined the regulatory effect of TTK on PI3K/AKT pathway, and found that the expression of p‐AKT in TCL was reduced after TTK knockdown (Figure 7E). While the relative expression level of p‐PI3K showed no remarkable change. The membrane aggregation and activation of AKT play an important role in the regulation of downstream molecules, for which IF staining was utilized to observe the effect of TTK knockdown on p‐AKT expression and cellular localization. It was found that TTK knockdown also reduced the intensity of p‐AKT on membrane, indicating the downregulation of p‐AKT activity in TCL after TTK knockdown (Figure 7F). In order to further explore molecular mechanism for synergetic effects, HDOCK was utilized to identify potential interactions between TTK and key molecules in the PI3K/AKT pathway. The potential interaction between TTK and AKT was disclosed with a Confidence Score of 0.7720 in the best model (Figure 7G, Docking score = −210.98, Ligand rmsd = 59.36). Co‐IP results further revealed the interaction between TTK and AKT in TCL cells (Figure 7H). The co‐localization of TTK and AKT in TCL cells was found using IF co‐staining (Figure 7I).

Furthermore, in‐vivo results revealed that both CFI‐402257 and Duvelisib significantly reduced the tumor growth rate, size and weight in TCL xenograft mouse models, and the combinational therapy of two drugs exerted enhanced inhibitory effect compared with single drug (Figure 7J–M). Overall, the above results supported that CFI‐402257 had synergistic anti‐TCL effect with Duvelisib in vitro and in vivo, and the synergistic effect may be mediated by the interaction and regulation of TTK on AKT.

## 3 Discussion

Our study illuminated the regulatory role and mechanism of TTK in the development of TCL. Association between high TTK expression and worse prognosis in TCL patients was revealed. Inhibition of TTK, by either knockdown or specific inhibitor CFI‐402257, exerted anti‐tumor effects in vitro and in vivo. Mechanically, targeting TTK induced autophagy through dephosphorylating p38α at Thr180/Tyr182, which further promoted the activation of AMPK/mTOR pathway.

It was recently reported that high expression level of TTK was correlated with the progression, clinical features and prognosis in various tumors.^[^ ^31^, ^32^ ^]^ TTK exhibited significant high expression in lung cancer and ovarian cancer, and was associated with Cisplatin resistance.^[^ ^27^, ^33^ ^]^ Meanwhile, TTK was also found to be up‐regulated in mantle cell lymphoma and diffuse large B‐cell lymphoma (DLBCL), and was associated with higher Ki‐67 index.^[^ ^34^ ^]^ However, the expression level and clinical prognostic value of TTK have not been reported in TCL. Here, our study found that the expression level of TTK was elevated in TCL patients, and high expression of TTK was associated with poor OS and PFS, suggesting that TTK could serve as a potential prognostic biomarker for TCL patients. Studies containing more patients and longer follow‐up time are still needed to further verify the prognostic role and clinical value of TTK in TCL.

Previous studies have demonstrated the oncogenic role of TTK in several solid tumors. Inhibition of TTK has been found to suppress the proliferation of tumor cells in lung cancer, colon cancer and breast cancer.^[^ ^35^, ^36^, ^37^, ^38^, ^39^ ^]^ Moreover, TTK inhibition could induce mitotic errors and enhance apoptosis in breast and pancreatic cancer.^[^ ^36^, ^39^ ^]^ In HCC, targeted inhibition of TTK reduced tumor aggressiveness, and induced DNA damage, micronuclei formation, autophagy, and cellular senescence.^[^ ^16^ ^]^ TTK has also been reported to be associated with drug resistance of tumor cells. Downregulation of TTK has been found to increase the sensitivity of tumor cells to Temozolomide, Cisplatin and Sorafenib in Glioblastoma (GBM) and colon cancer.^[^ ^27^, ^40^ ^]^ Furthermore, recent studies have reported the relevance of TTK to tumor immune microenvironment, where TTK inhibition could promote T cell‐mediated anti‐tumor immunity and enhance the sensitivity to PD‐1 blockade therapy.^[^ ^41^, ^42^ ^]^ In terms of hematologic malignancy, TTK inhibition was found to induce chromosome segregation errors, DNA damage, and micronuclei formation in acute myeloid leukemia (AML).^[^ ^43^ ^]^ However, the biological role of TTK in TCL has not yet been reported. Our study found that TTK knockdown exerted inhibitory effect on TCL progression by suppressing cell viability and proliferation, inducing G2/M cell cycle arrest, promoting DNA damage and activating apoptosis. These results suggested that TTK might play a pro‐tumorigenic role in TCL, indicating its potential as therapeutic target in TCL treatment.

As a specific inhibitor of TTK, CFI‐402257 has been found to exert significant anti‐tumor effects in solid tumors, such as GBM, malignant mesothelioma, and lung cancer, by competitively inhibiting the kinase activity of TTK.^[^ ^40^, ^44^ ^]^ Previous studies have reported that CFI‐402257 activated the cGAS‐STING pathway by promoting micronuclei formation, and enhanced the efficacy of anti‐PD‐1 treatment in KRAS‐LKB1 mutant lung cancer.^[^ ^45^ ^]^ CFI‐402257 exhibited anti‐tumor effects in in vivo and in vitro models of HCC and induced senescence‐associated secretory phenotype through activating the DDX41‐STING pathway.^[^ ^16^ ^]^ Moreover, CFI‐402257 was found to display enhanced anti‐tumor effects in ER^+^ breast cancer with resistance to CDK4/6 inhibitors.^[^ ^15^ ^]^ Clinical trials assessing CFI‐402257 included monotherapy in advanced cancer patients (NCT02792465, NCT05251714), in combination with fulvestrant in ER and/or PR‐positive breast cancer patients (NCT03568422), and in combination with paclitaxel in ER+/HER2‐advanced breast cancer patients (NCT05251714). According to the interim results of NCT03568422, 47% (8/17) of patients who received CFI‐402257 in combination with paclitaxel achieved clinical benefit (complete response or partial response or stable disease). Notably, CFI‐402257 has received FDA fast‐track designation, indicating the clinical application potential of CFI‐402257 for tumor treatment. Here, our results demonstrated that CFI‐402257 exerted anti‐tumor effects in TCL cells and xenograft mouse models, providing a promising strategy for the treatment of TCL.

TTK, as a bispecific protein kinase, has been reported to be involved in cell proliferation and division processes during tumor progression.^[^ ^46^ ^]^ For example, TTK was found to phosphorylate tyrosine kinase c‐Abl at Thr735, and the knockdown of TTK suppressed the growth of breast cancer cells by downregulating the phosphorylation of c‐Abl.^[^ ^7^ ^]^ Besides, TTK also promoted the phosphorylation of E3 ubiquitin ligase MDM2, thereby promoting DNA oxidative damage repair and cell survival.^[^ ^8^ ^]^ In our study, we identified that p38α was a downstream kinase substrate of TTK, and TTK could directly interact with p38α in TCL cells. p38α, the central component of p38‐MAPK pathway, has been found to regulate various cellular functions, including cell proliferation, differentiation, stress response, apoptosis, cell migration and autophagy, through phosphorylating downstream transcription factors.^[^ ^47^ ^]^ In breast cancer, p38α knockout induced the DNA damage response, increased replication stress, and chromosomal instability.^[^ ^48^ ^]^ In osteosarcoma, p38α activation triggered the formation of autophagosomes and enhanced basal autophagy flux, which in turn induced tumor cells to enter senescence instead of apoptosis.^[^ ^30^ ^]^ Several studies have shown that p38α exerts regulating effect on ubiquitin ligases. As previously reported, phosphorylation of p38α promoted its K48‐linked polyubiquitination mediated by the E3 ubiquitin ligase Nedd4.^[^ ^49^ ^]^ P38α has been reported to inhibit the polyubiquitination activity of Ube3c in response to progesterone by phosphorylating serine 741 of Ube3c.^[^ ^24^ ^]^ In differentiated muscle cells, activation of p38α is involved in the regulation of EZH2 levels via the E3 ubiquitin ligase Praja1.^[^ ^25^ ^]^ In our study, we found that TTK could regulate the expression and activation of p38α in TCL by phosphorylating it at Thr180/Tyr182 and then inhibiting its ubiquitin‐proteasomal degradation coupled with dephosphorylation. Further results indicated that TTK modulated the autophagy of TCL cells through phosphorylating p38α. Moreover, further exploration is still needed to investigate the relevant enzymes and target sites involved in ubiquitination regulation of p38α by TTK in TCL.

Additionally, AMPK and its key downstream molecule mTOR play a significant role in maintaining cellular energy homeostasis and cell growth.^[^ ^50^, ^51^ ^]^ Previous studies have shown that p‐p38α regulated gluconeogenesis as a negative regulator of AMPK signaling.^[^ ^52^ ^]^ The dysregulation of the AMPK/mTOR pathway was also found in lymphoma,^[^ ^53^ ^]^ which has been considered as a potential target for lymphoma treatment. In T‐ALL, metformin could suppress tumor growth by activating AMPK,^[^ ^54^ ^]^ and the AMPK inhibitor compound C could induce apoptosis in HTLV‐1‐infected T cell lines through promoting DNA damage.^[^ ^55^ ^]^ Meanwhile, it has been proposed that genomic instability generated micronuclear structures could trigger autophagy degradation,^[^ ^56^ ^]^ Activation of Ataxia‐telangiectasia mutated proteins (ATM) by DNA damage induced the activation of AMPK and further abrogated the inhibitory effect of TORC1 on autophagy.^[^ ^57^ ^]^ In addition, the HDACi valproic acid (VPA) has been found to induce autophagy in T‐ALL through the activation of AMPK/mTOR pathway.^[^ ^58^ ^]^ Recent research found that malate metabolizing enzyme ME2 stimulated mTOR complex 1 activity to promote the development of TCL, while mTOR inhibitor Rapamycin effectively inhibited the growth of TCL in vivo.^[^ ^59^ ^]^ The mTOR inhibitor Rapamycin has also shown clinical activity in mantle cell lymphoma.^[^ ^60^ ^]^ However, limited research on its clinical effects in TCL has been reported. Here, our results revealed that TTK regulated the AMPK/mTOR pathway through p38α during the development of TCL. Besides, we disclosed that Rapamycin had inhibitory effect on TCL proliferation, which could be enhanced by the TTK inhibitor CFI‐402257. The precise regulatory mechanism of TTK in the regulation of AMPK/mTOR pathway and the potential of combining CFI‐402257 and Rapamycin in TCL treatment remain to be further explored.

Moreover, given the important role of PI3K in promoting tumor progression, Duvelisib, a dual inhibitor targeting PI3Kγ/δ, has been indicated to exhibit anti‐tumor effects in tumors with predominant expression of PI3Kγ/δ, especially in B‐cell and T‐cell lymphoma.^[^ ^61^ ^]^ The results of phase 2 clinical trial for Duvelisib showed that the objective response rate (ORR) of monotherapy for refractory PTCL was 49%, indicating the promising application prospect of Duvelisib in TCL.^[^ ^62^ ^]^ Here, the potential combined anti‐TCL effect of Duvelisib with CFI‐402257 was discovered through drug library screening and further in vitro and in vivo study found that CFI‐402257 exerted stable synergistic effect with Duvelisib, which might be mediated by the interaction between AKT and TTK. Overall, our finding might provide a promising novel combination therapy strategy for TCL patients, and further research on key problems, such as the optimal dosage, timing of administration and adverse reactions, is still needed to evaluate the clinical potential of Duvelisib and CFI‐402257 combined therapy strategy for TCL treatment.

Considering the limitations of this study, our functional experiments incorporated TCL cells including T‐ALL, anaplastic large cell lymphoma and cutaneous T‐cell lymphoma. Due to the heterogeneity of molecular features and clinical manifestations among TCL subtypes, the variability of the biological function and regulatory mechanisms for TTK in cells of different TCL subtypes still needs to be further explored. Meanwhile, recent studies have shown that the promoter of TTK is regulated by H3K18 lactylation in pancreatic cancer,^[^ ^63^ ^]^ the regulatory mechanisms of upstream transcription and activation of TTK in TCL remains to be further investigated.

Collectively, our results revealed the elevated expression, prognostic value and oncogenic role of TTK in TCL, and highlighted the therapeutic potential of CFI‐402257 in TCL treatment. Notably, inhibiting TTK exerted anti‐TCL effects in vitro and in vivo through downregulating the phosphorylation and upregulating the ubiquitination of p38α, which further promoted autophagy and the activation of the AMPK/mTOR pathway. Overall, these findings identified TTK as a novel target for TCL treatment and underscored the potential of CFI‐402257 as promising therapeutic strategy for TCL, which might contribute to the targeted therapy and prognosis improvement in TCL patients.

## 4 Experimental Section

### Sex as a Biological Variable

In this study, sex was not considered as a biological variable.

### Patient Samples

This study was approved by the Medical Ethical Committee of Shandong Provincial Hospital and written informed consent in accordance with the Declaration of Helsinki was obtained from each patient. Paraffin‐embedded archived samples were collected from 92 newly diagnosed TCL patients and 40 reactive hyperplasia patients from 2012 to 2023. Histological diagnoses were established according to the 2022 WHO classification.^[^ ^64^ ^]^ All enrolled TCL patients have received standard treatment according to international guidelines. Details of patients were provided in Table S1 (Supporting Information). Normal T cells were purified from freshly isolated Peripheral blood mononuclear cells (PBMCs) from healthy donors by double‐rosetting with 2‐aminoethylosothiouronium bromide‐treated sheep red blood cells (SRBCs).^[^ ^65^ ^]^

### Cell Lines

Karpas‐299, Jurkat, Molt‐3 and Myla 3676 cell lines were purchased from ATCC, cultured in IMDM (Gibco, CA, USA) supplemented with 10% fetal bovine serum (HyClone, UT, USA), 1% penicillin/streptomycin mixture, and 2mM glutamine, and incubated at 37 °C in humidified air containing 5% CO_2_. All cells were examined for short tandem repeat (STR) and mycoplasma infection periodically.

### Reagents

CFI‐402257 (GC18491) and Dorsomorphin (GC17243) was obtained from GlpBio (GlpBio, CA, USA). Duvelisib (T1988), MHY1485 (T1908) were bought from Topscience (Topscience, Shanghai, China). FDA Anti‐tumor Drug Library was purchased from Selleck Chemicals (L8000, Selleck Chemicals, TX, USA). MG132 (HY‐13259), CHX (HY‐12320), and Chloroquine (HY‐17589A) were obtained from MedChemExpress (MedChemExpress, NJ, USA).

### Bioinformatics Analysis

The mRNA expression microarray and prognosis data of TCL patients and healthy donors were derived from the public database GEO (https://ww.ncbinlm.nih.gov/geo). The data from GSE45712 (n = 117) and GSE6338 (n = 51) were used for differentially expressed genes (DEGs) analysis. The sva package in R was utilized to eliminate potential batch effects across different datasets. The limma package in R was used to identify DEGs in TCL clinical samples and normal T cells from healthy donors (P < 0.05, fold change>1.2). Functional enrichment was performed based on the KEGG and GO databases using clusterProfiler in R. CRISPR Screen data of TCL cell lines was obtained from public database BioGRID ORCS (https://orcs.thebiogrid.org/).

### Cell Transfection

Lentivirus vectors encoding short hairpin TTK (Sh‐TTK), TTK overexpression (LV‐TTK) or control were from Genechem (Shanghai, China), and plasmids, including TTK‐Flag/His, TTK‐Flag/His (K553A), TTK‐Flag/His (1‐192aa), TTK‐Flag/His (193‐524aa), TTK‐Flag/His (525‐857aa), p38α‐HA, p38α‐HA (T180E/Y182E), p38α‐HA (T180A/Y182A) were synthesized by Biosune (Shanghai, China).

### IHC and Hematoxylin–Eosin (HE) Staining

IHC and HE staining were performed according to standard methods.^[^ ^66^ ^]^ Reactive hyperplasia of lymph nodes was considered as control group. Staining results were evaluated by two independent observers who were blinded to patients’ clinical data at two different time points. IHC score was calculated by the accumulation of multiplying proportion score (0, none; 1, 1–25%; 2, 26–50%; 3, 51–75%; and 4, 76–100%) and intensity score (1, negative; 2, weak; 3, moderate; and 4, strong). Scores of 0–5 were defined as negative expression, 6–16 as positive expression. The primary antibodies included TTK (10381‐1‐AP, Proteintech, Wuhan, China) and Ki‐67 (27309‐1‐AP, Proteintech).

### Western Blotting

Cell lysates were extracted using RIPA buffer (Pierce) together with inhibitors for proteases and phosphatases (PhosSTOP, Roche, Basel, Switzerland). Following the previous description,^[^ ^67^ ^]^ WB analysis was conducted. The primary antibodies were as follows, TTK (A2500, Abclonal, Wuhan, China), p38α (66234‐1‐Ig, Proteintech), Caspase‐3 (ab184787, Abcam, MA, USA), cleaved‐Capase‐3 (68773‐1‐Ig, Proteintech), Caspase‐9 (10380‐1‐AP, Proteintech), cleaved‐Caspase‐9 (9509, Cell Signaling Technology (CST), MA, USA), PARP (13371‐1‐AP, Proteintech), LC3B (ab192890, Abcam), phospho‐p38α (Thr180/Tyr182) (4511, CST), AKT (60203‐2‐Ig, Proteintech), phospho‐AKT (2535, CST), AMPK (ab32047, Abcam), phospho‐AMPK (2535, CST), mTOR (2983, CST), phospho‐mTOR (5536, CST), GAPDH (10381‐1‐AP, Proteintech), p62 (ab207305, Abcam), Beclin (ab207612, Abcam), c‐myc (18583, CST), p‐H2AX (9718, CST), Ubiquitin (3936, CST), HA (3724, CST), TSC2 (24601‐1‐AP, Proteintech), p‐TSC2 (5584, CST), Raptor (20984‐1‐AP, Proteintech), phospho‐Raptor (2083, CST). Following overnight incubation with primary antibodies at 4 °C, membranes were washed with TBS‐T and then treated with HRP‐conjugated secondary antibody (Zhongshan Goldenbridge). Images were captured with the Amersham Imager 680 imaging system (General Electric, USA).

### Cell Proliferation and Viability Assay

Cell proliferation was assessed using the Cell Counting Kit‐8 (CCK8) assay kit (CK04, DOJINDO, Japan) and Multiskan GO Microplate Reader (Thermo Scientific, IL, USA). Cell viability was evaluated using CTG assays (G7570, Promega Corporation, WI, USA) and luminescence was recorded with a microplate luminometer (Centro XS3 LB960, Berthold Technologies, Stuttgart, Germany).

### Flow Cytometry Analysis

For apoptosis analysis, TCL cells were harvested and washed twice with cold PBS and resuspended cells in 1× binding buffer, followed by adding 5 µL of Annexin V‐PE and 5 µL of 7‐AAD (559763, BD Biosciences, USA). For cell cycle analysis, the 70% ethanol fixation at −20 °C overnight was followed by washing with PBS and staining with PI/RNAse stain (550825, BD Biosciences) for 15 min. Flow cytometry was used to analyze cells after incubation in the dark for 15 min. Cell cycle and apoptosis were determined by flow cytometric analysis on a Navios Flow Cytometer (Beckman Coulter, CA, USA).

### Quantitative Proteomic Analysis of Phosphorylation Modification

TTK knockdown and control Jurkat cells were centrifuged at 1000 rpm for 5 min, then washed three times with pre‐cooled PBS solution. Cells were collected after centrifuging at 1000 rpm for 5 min. Phosphoproteomic quantification was performed by Novogene (Beijing, China).

### Molecular Docking

Protein structures were obtained from the PDB database (https://www.rcsb.org/). The HDOCK online server (http://hdock.phys.hust.edu.cn/) was used for molecular docking. HDOCK was also utilized to analyze protein‐protein docking with different conformations, binding activities under various conformations, and amino acid residues interacting within 5Å. The PyMOL (version 4.6.0) software was employed to illustrate the amino acid residues involved in the interaction between the two proteins and to measure hydrogen bond distances.

### Co‐IP

Protein extraction and purification were performed using Pierce Co‐Immunoprecipitation Kit (26149, Thermo Fisher Scientific). The primary antibodies included TTK (A2500, Abclonal), p38α (66234‐1‐Ig, Proteintech), AKT (60203‐2‐Ig, Proteintech), Flag (66008‐4‐Ig, Proteintech), Rabbit Control IgG (AC005, Abclonal), Mouse Control IgG (AC011, Abclonal).

### CHX Chase Assay

TCL cells were incubated with 20µM MG132 (proteasomal inhibitor), or left untreated. After the treatment of 50 µg mL^−1^ CHX for different time, cells were harvested and prepared for WB.

### IF Assay and Confocal Microscopy

Confocal microscopy was performed using the Leica TCS SP8 MP confocal microscope system (Germany). The primary antibodies included TTK (10381‐1‐AP, Proteintech), p38α (66234‐1‐Ig, Proteintech), AKT (60203‐2‐Ig, Proteintech), phospho‐AKT (2535, CST), p‐H2AX (9718, CST), LC3B (14600‐1‐AP, Proteintech), α‐Tubulin (11224‐1‐AP, Proteintech).

### In Vivo Xenograft Mouse Models

This study was approved by the Animal Care and Research Advisory Committee of Shandong Provincial Hospital and guidelines of it were strictly followed in all animal experiments. No blinding was performed. The 4‐week‐old SCID beige female mouse were randomized (simple randomization) into groups and 1 × 10^7^ Karpas‐299 cells (Blank, Sh‐Control, Sh‐TTK) were subcutaneously injected into their right hind legs. CFI‐402257 and Duvelisib were dissolved in 90% PEG400 and 10% water, and daily administered by gavage for a week at a concentration of 6 and 10mg kg^−1^, respectively. The animals were imaged using an in vivo small animal imaging system (PerkinElmer, USA).

### Statistical Analysis

All calculations were performed using SPSS 23.0 (SPSS Inc., USA) and R. Each in vitro experiment was repeated three times and experimental data were depicted as mean ± standard deviation (SD). Data were tested for homogeneity of variances and normality. Quantitative variables were analyzed using Students t‐test and non‐parametric tests while categorical variables were analyzed by χ2‐tests. Kaplan–Meier analysis was performed for survival curves and the difference between survival curves was compared using the Log‐rank test. There was no statistical method used to determine the sample size in our study. Differences were considered statistically significant at p < 0.05 (*p < 0.05, **p < 0.01, ***p < 0.001).

## Ethics Approval and Consent to Participate

This study was approved by the Medical Ethical Committee of Shandong Provincial Hospital and written informed consent in accordance with the Declaration of Helsinki was obtained from each patient.

## Conflict of Interest

The authors declare no conflict of interest.

## Author Contributions

B.Y.L. collected clinical data. B.Y.L., T.G.L., M.F.D. and X.L.Z. wrote and edited this manuscript and created figures and tables. B.Y.L., Y.Y.J., J.J.S., W.Y.S. performed research and analyzed the data. X.X.Z., X.W. and S.F.H. reviewed and revised the manuscript. All authors read and approved the final manuscript.

## Supporting information



Supporting Information

## Data Availability

The data that support the findings of this study are available from the corresponding author upon reasonable request.
