# Supplementary material for: Targeting TTK Inhibits Tumorigenesis of T‐Cell Lymphoma Through Dephosphorylating p38α and Activating AMPK/mTOR Pathway
Source: Adv Sci (Weinh). 2025 Jan 21;12(10):2413990. doi: 10.1002/advs.202413990 (PMC11905054; doi:10.1002/advs.202413990)
Supplement: Supplementary file 1 — Supporting Information [file ADVS-12-2413990-s001.pdf]

ADVANCED SCIENCE
Open Access

Supporting Information

for *Adv. Sci.*, DOI 10.1002/advs.202413990

Targeting TTK Inhibits Tumorigenesis of T-Cell Lymphoma Through Dephosphorylating p38α and Activating AMPK/mTOR Pathway

Bingyu Liu, Tiange Lu, Mengfei Ding, Xiaoli Zhou, Yujie Jiang, Juanjuan Shang, Wenyue Sun, Shunfeng Hu, Xin Wang* and Xiangxiang Zhou**

Figure S1

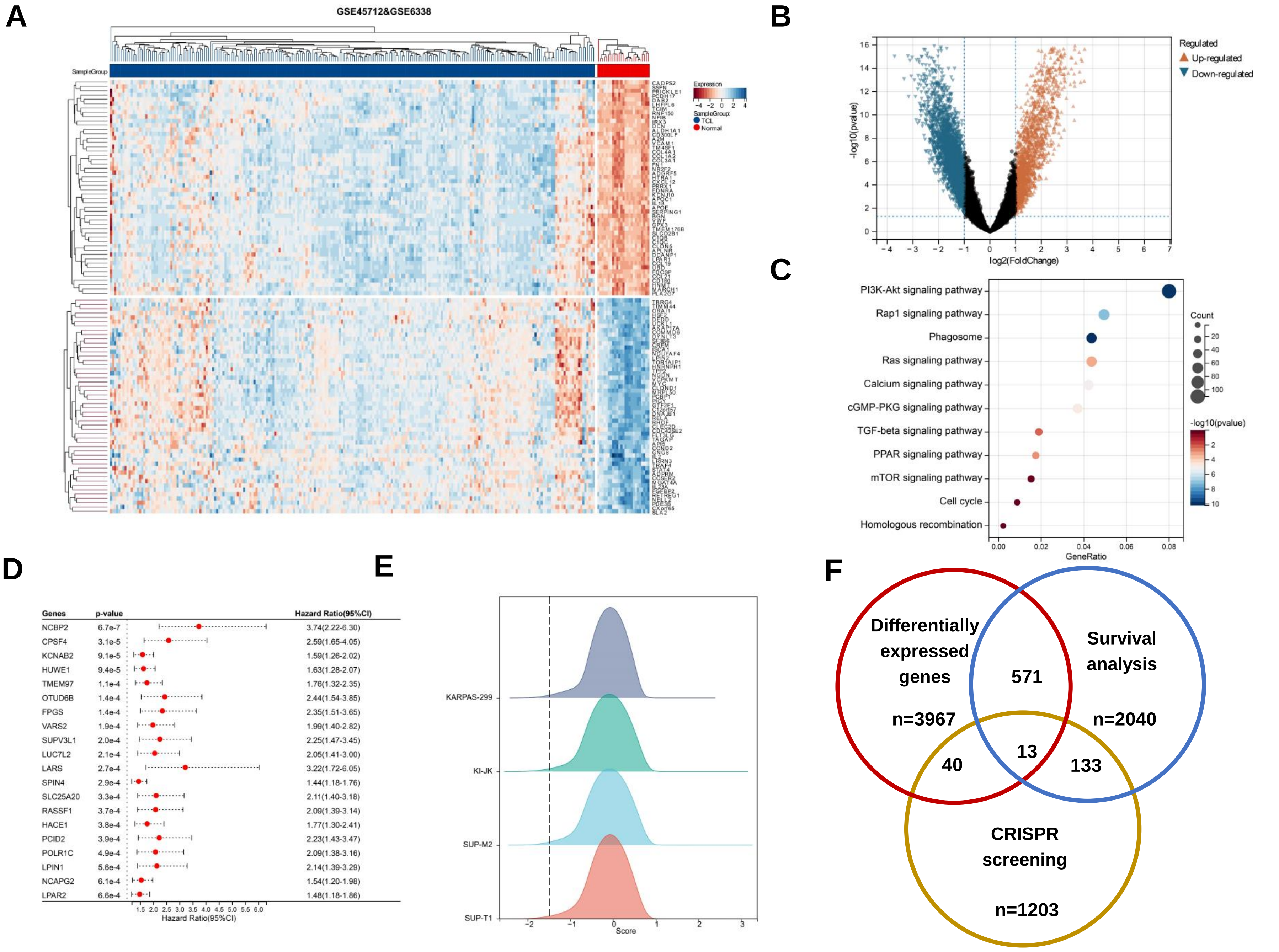

Figure S1. Comprehensive analysis of key driver genes in TCL. A. Heatmap illustrated the top 50 differentially expressed genes in TCL tissues compared to normal T cells by merging GSE45712 (n=117) and GSE6338 (n=51) datasets (P<0.05). **B.** Volcano plot displayed the Fold change and P value distribution of differentially expressed genes in TCL. **C.** The KEGG enrichment analysis revealed associated enriched pathways of DEGs in TCL. **D.** Forest plot showed the hazard ratio, 95% confidence interval, and P values of the top 20 genes significantly associated with poor prognosis in TCL patients from GSE58445 dataset (n=192). **E.** Mountain plot depicted the distribution of CRISPR screening scores for all genes across different TCL cell lines. **F.** Through integrating DEGs, survival analysis, and CRISPR screening, 13 key genes in the progression of TCL were identified (TTK, CDCA8, BUB1B, HJURP, HIST1H2BM, TICAM2, KIF23, CDT1, RPP40, CRCP, SDC1, HAMP, RAD51).

Figure S2

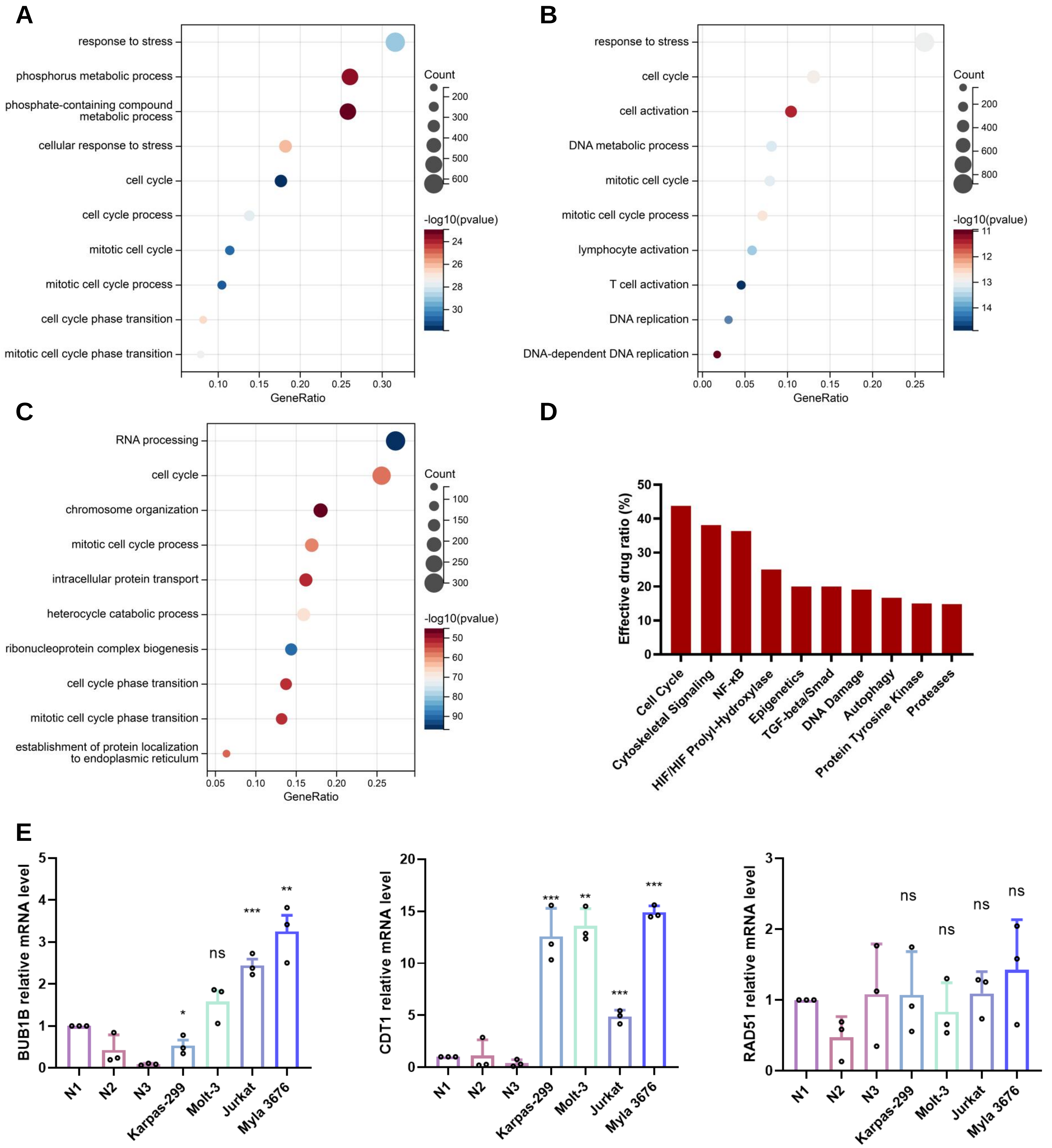

Figure S2. Cell cycle regulation was associated with the development of TCL. A. The GO enrichment analysis revealed associated enriched biology processes of DEGs in TCL. **B.** The GO enrichment analysis of prognostic-associated genes in TCL. **C.** The GO enrichment of survival-critical genes in TCL. **D.** Bar chart showed the top ten drug types from the FDA Anti-tumor Drug Library with the highest effective inhibition ratios in Jurkat cell line. **E.** qPCR analysis demonstrated the relative mRNA expression levels of BUB1B, CDT1, and RAD51 in TCL cells and healthy donor T cells. Data are shown as the mean ± SD. *P < 0.05; **P < 0.01; ***P < 0.001.

Figure S3

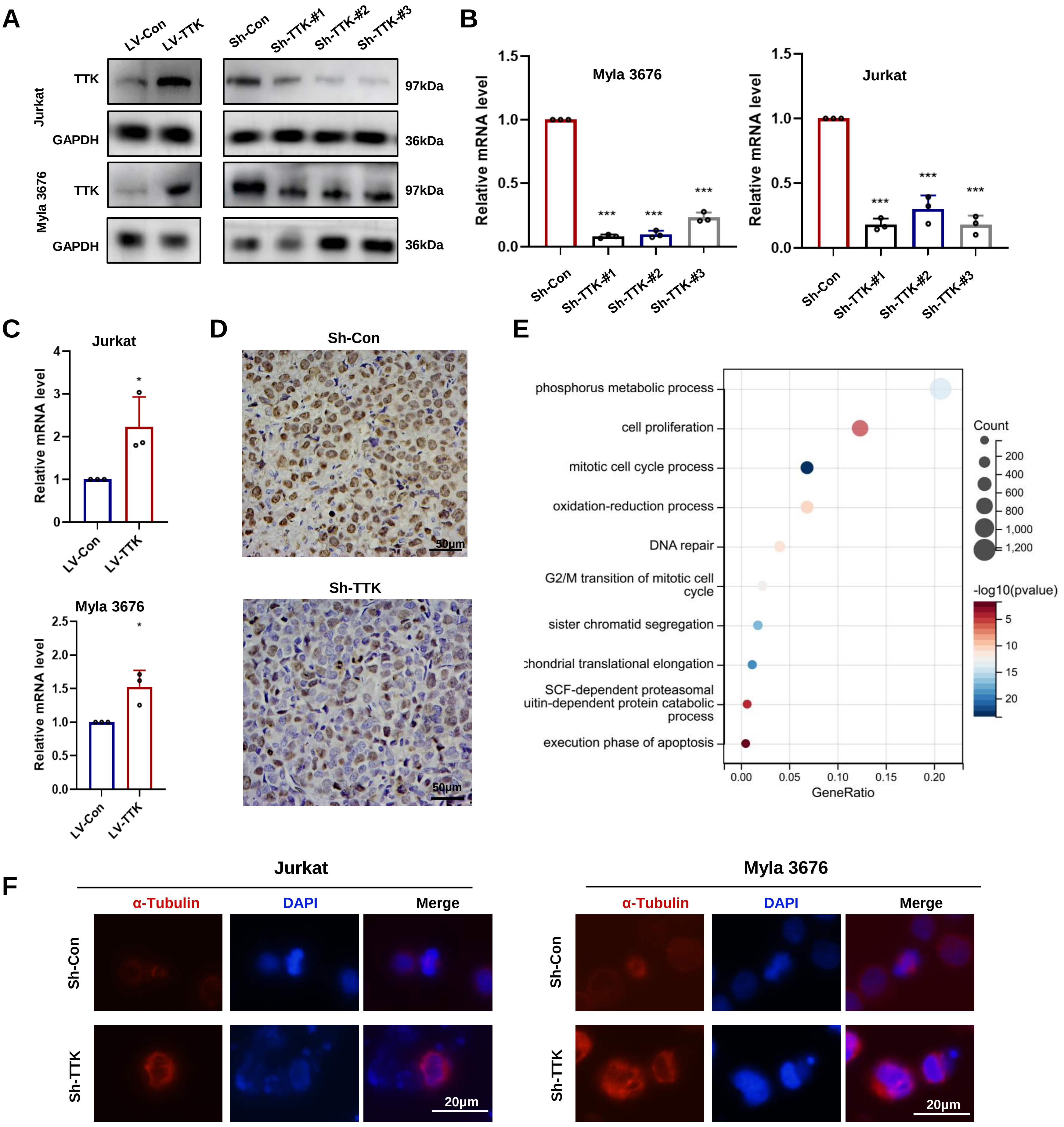

Figure S3. **TTK was associated with tumor progression in TCL. A.** Relative expression of TTK protein were confirmed by WB in lentivirus transfected TCL cells. **B-C.** Relative expression of TTK mRNA were confirmed by qPCR in lentivirus transfected TCL cells. **D.** IHC of ki-67 in Sh-Con and Sh-TTK TCL mouse models. Bar = 50 μm. **E.** GO analysis showed that TTK was associated with the proliferation, apoptosis, cell cycle, chromosome separation and DNA damage based on GSE58445. **F.** α-Tubulin immunofluorescence showed chromosome missegregation in TCL cells after TTK knockdown. Bar = 20 μm. Data are shown as themean ± SD. *P < 0.05; **P < 0.01; ***P < 0.001.

Figure S4

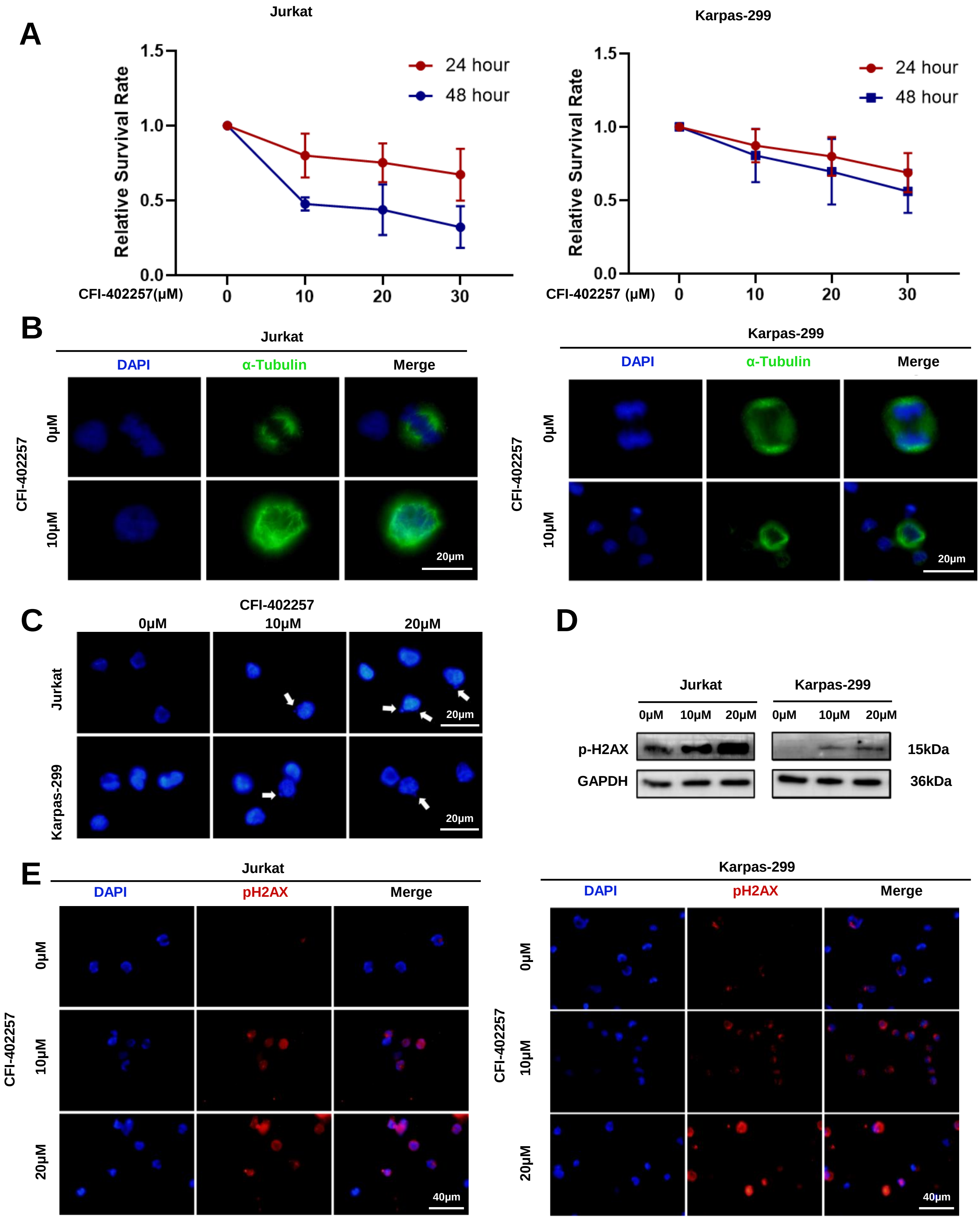

Figure S4. Targeting TTK by CFI-402257 regulated the cell cycle, chromosome separation, DNA damage and apoptosis in TCL cells. A. CFI-402257 exerted time- and dose-dependent inhibitory effect on the viability in TCL cells. **B.** α-Tubulin immunofluorescence showed chromosome missegregation in TCL cells after CFI-402257 treatment. **C.** CFI-402257 increased the number of micronuclei in TCL cells. **D**. CFI-402257 increased the expression level of p-H2AX in TCL cells. **E.** CFI-402257 increased the intensity of p-H2AX in TCL cells. Bar = 40 μm.

Figure S5

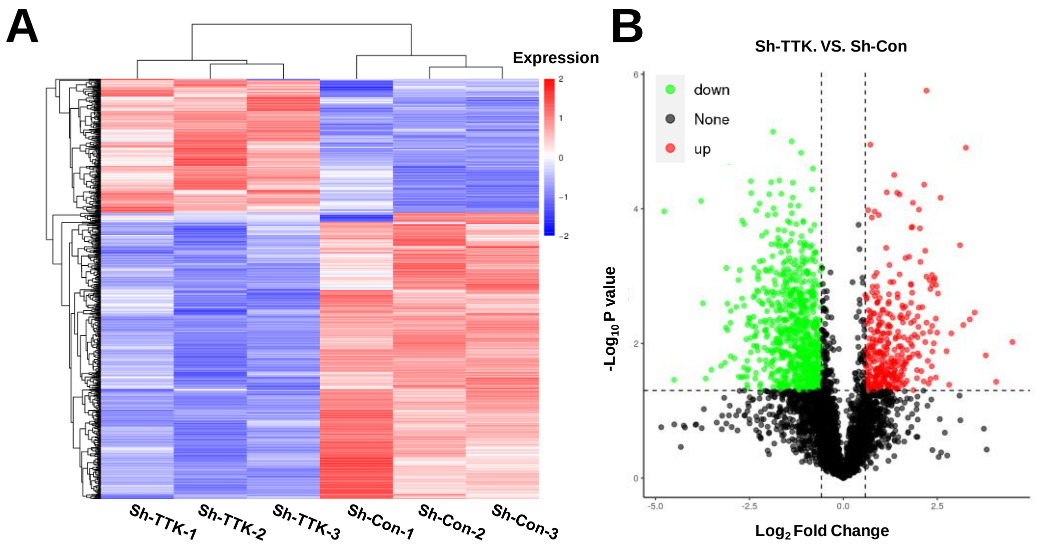

Figure S5. Phosphoproteomics quantification after TTK knockdown. A-B. Phosphoproteomics quantification in TCL cells identified 1129 differentially phosphorylated proteins after TTK knockdown.

Figure S6

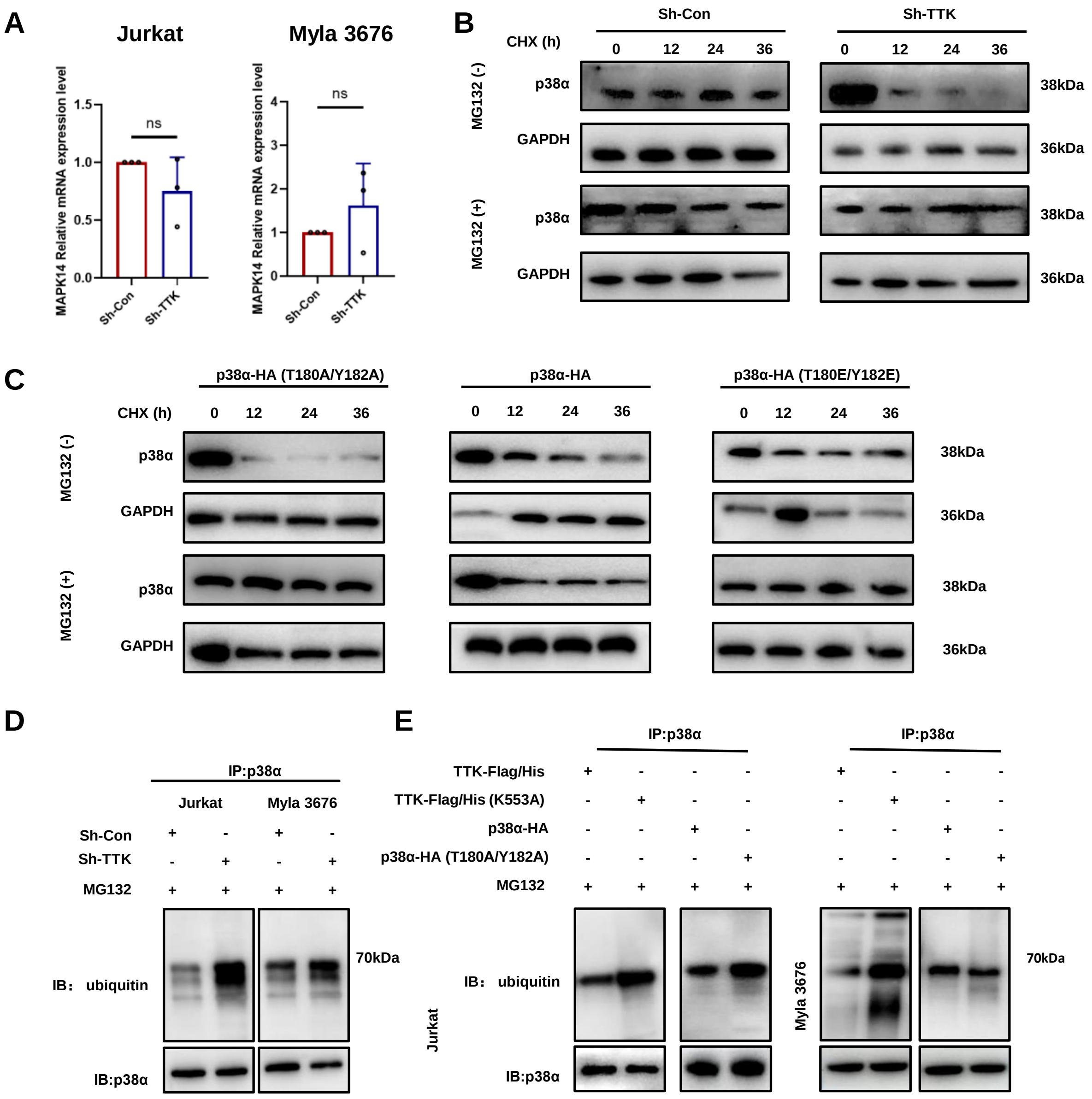

Figure S6. TTK enhanced the de-ubiquitination of p38α through phosphorylation. A. TTK knockdown did not decrease the mRNA expression of p38α. **B.** TTK knockdown promoted the lysosomal degradation of p38α. **C.** Phosphorylation inactivation of p38α promoted the lysosomal degradation of p38α and phosphorylation activation of p38α inhibited it. **D.** TTK knockdown promoted p38α ubiquitination. **E.** TTK kinase inactivation and p38α phosphorylation inactivation promoted p38α ubiquitination.

Figure S7

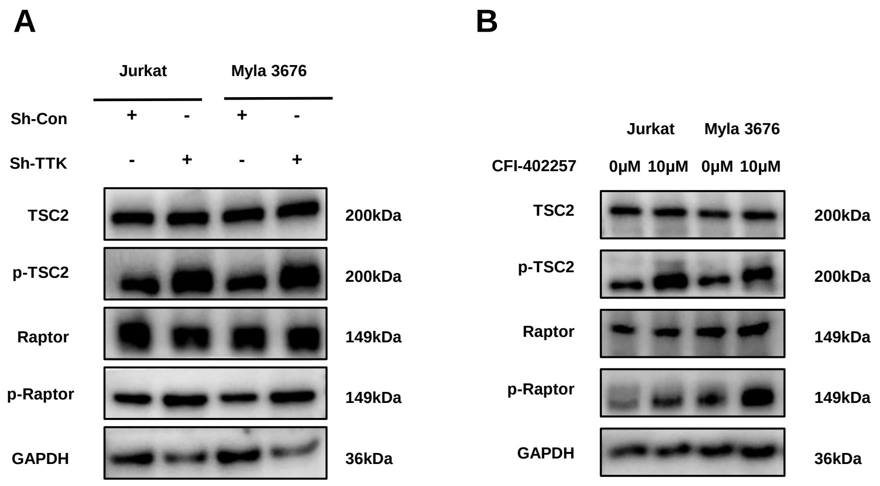

Figure S7. TTK inhibition elevated the expression of p-TSC2 and p-Raptor. A. WB analysis showed the expression level of p-TSC2 and p-Raptor after TTK knockdown. **B.** WB analysis showed the expression level of p-TSC2 and p-Raptor after CFI-402257 treatment (10μM, 48 h).

Figure S8

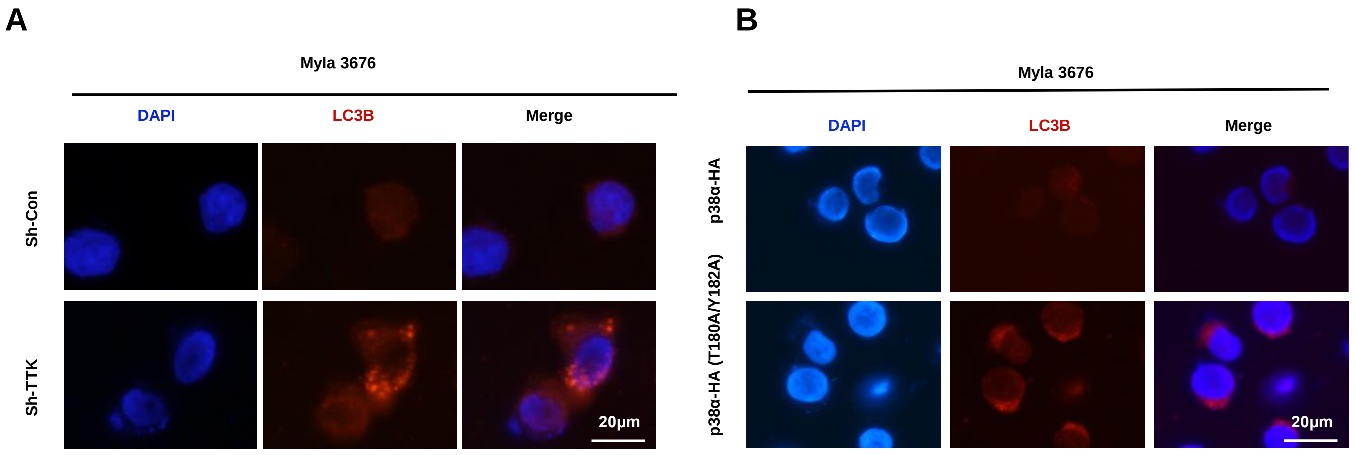

Figure S8. TTK inhibition and p38α phosphorylation inactivation enhanced autophagy in TCL. A. Immunofluorescence analysis of LC3B autophagic vesicles (red) after TTK knockdown in TCL cells. Bar = 20 μm. **B.** Immunofluorescence analysis of LC3B autophagic vesicles (red) after transfection with p38α phosphorylation inactivation plasmid in TCL. Bar = 20 μm.

Figure S9

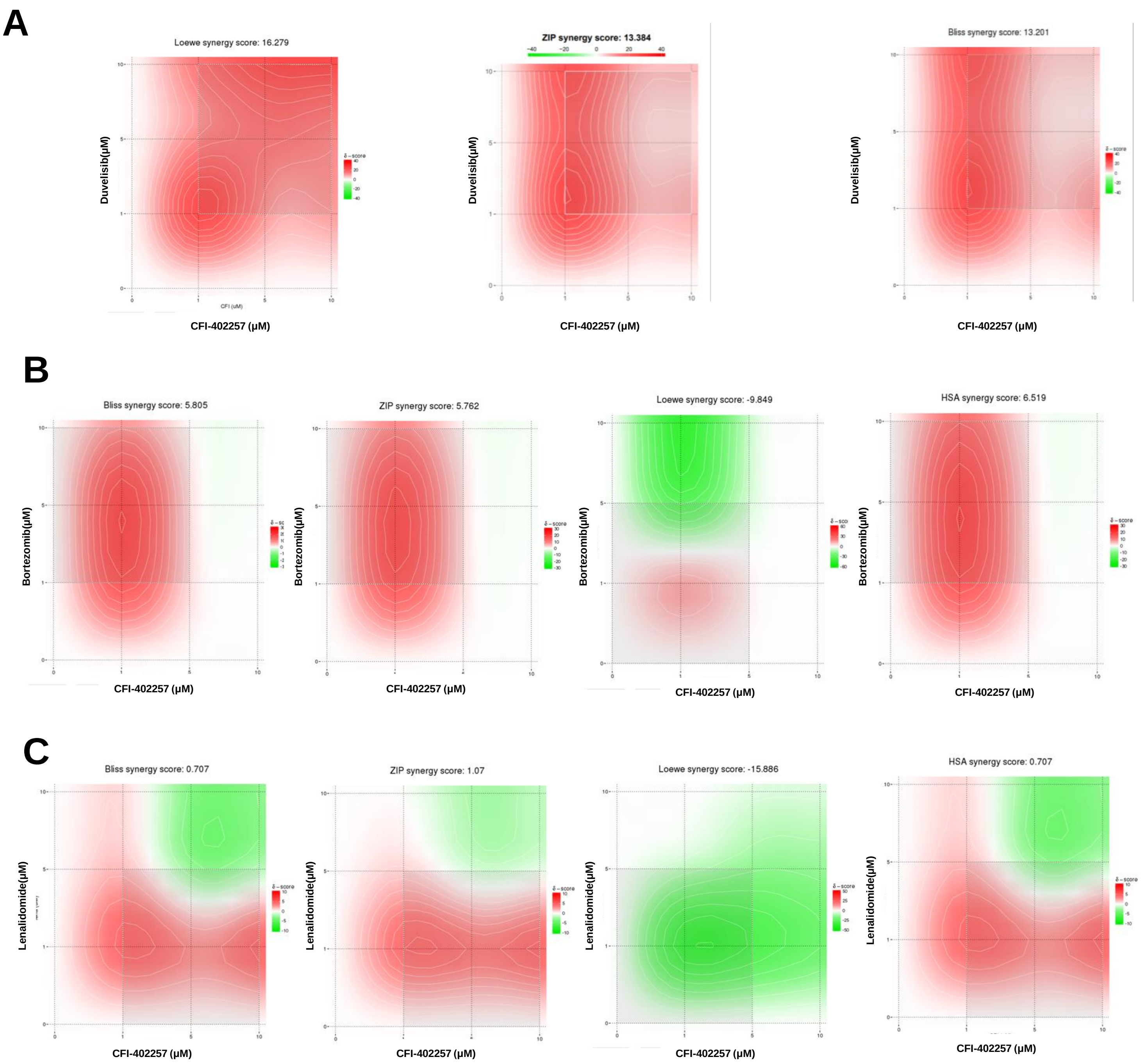

Figure S9 Synergy scores of CFI-402257 with anti-TCL drugs. A-C. Bliss, ZIP, Loewe, and HSA Synergy score of Duvelisib, Bortezomib, and Lenalidomide in combination with CFI-402257.

Table S1. Clinical characteristics based on TTK expression in TCL patients

Characteristics	Positive (N=32)	Negative (N=59)	Total(N=91)	P value
**Gender**				0.89
Female	9(11.25%)	20(25.00%)	29(36.25%)	
Male	18(22.50%)	33(41.25%)	51(63.75%)	
**Age**				0.22
<60	16(20.00%)	40(50.00%)	56(70.00%)	
≥60	11(13.75%)	13(16.25%)	24(30.00%)	
**Subtype**				0.36
AITL	6(7.50%)	6(7.50%)	12(15.00%)	
ALCL-ALK+	6(7.50%)	8(10.00%)	14(17.50%)	
ALCL-ALK-	4(5.00%)	5(6.25%)	9(11.25%)	
PTCL-NOS	3(3.75%)	12(15.00%)	15(18.75%)	
NK/TCL	8(10.00%)	22(27.50%)	30(37.50%)	
**IPI score > 3**				0.2
No	14(20.00%)	37(52.86%)	51(72.86%)	
Yes	9(12.86%)	10(14.29%)	19(27.14%)	
**Ann Arbor Stage**				0.08
I/II	6(7.50%)	24(30.00%)	30(37.50%)	
III/IV	21(26.25%)	29(36.25%)	50(62.50%)	
**B Symptoms**				0.53
Absence	11(13.75%)	27(33.75%)	38(47.50%)	
Presence	16(20.00%)	26(32.50%)	42(52.50%)	
**Liver Involvement**				0.63
No	21(26.25%)	45(56.25%)	66(82.50%)	
Yes	6(7.50%)	8(10.00%)	14(17.50%)	
**Spleen Involvement**				0.55
No	19(23.75%)	42(52.50%)	61(76.25%)	
Yes	8(10.00%)	11(13.75%)	19(23.75%)	
**BM Involvement**				0.16
No	22(27.50%)	50(62.50%)	72(90.00%)	
Yes	5(6.25%)	3(3.75%)	8(10.00%)	
**CNS Involvement**				0.73
No	26(32.50%)	53(66.25%)	79(98.75%)	
Yes	1(1.25%)	0(0.0e+0%)	1(1.25%)	
**Elevated LDH**				0.27
No	9(13.04%)	26(37.68%)	35(50.72%)	
Yes	14(20.29%)	20(28.99%)	34(49.28%)	
**Elevated β2-MG**				0.94
No	14(17.95%)	26(33.33%)	40(51.28%)	
Yes	12(15.38%)	26(33.33%)	38(48.72%)	
**Elevated CRP**				0.34
No	5(7.46%)	17(25.37%)	22(32.84%)	

Yes	17(25.37%)	28(41.79%)	45(67.16%)	
**Elevated SA**				**0.01**
No	9(12.50%)	34(47.22%)	43(59.72%)	
Yes	15(20.83%)	14(19.44%)	29(40.28%)	
**EB Virus Infection**				0.92
No	7(13.73%)	10(19.61%)	17(33.33%)	
Yes	12(23.53%)	22(43.14%)	34(66.67%)	
**Ki-67**	60(20-90)	70(0-95)	60(0-95)	0.6
**Chromosomal Abnormalities**				1
No	2(40.00%)	1(20.00%)	3(60.00%)	
Yes	2(40.00%)	0(0.0e+0%)	2(40.00%)	
**Treatment**				0.31
CT	5(6.41%)	16(20.51%)	21(26.92%)	
CT+BV	2(2.56%)	3(3.85%)	5(6.41%)	
CT+HDACi	6(7.69%)	6(7.69%)	12(15.38%)	
CT+RT	0(0.0e+0%)	4(5.13%)	4(5.13%)	
CT+TRANS	2(2.56%)	1(1.28%)	3(3.85%)	
N	11(14.10%)	18(23.08%)	29(37.18%)	
RT	0(0.0e+0%)	4(5.13%)	4(5.13%)	
**Therapeutic Efficacy**				0.92
SD+PD	11(23.40%)	28(59.57%)	39(82.98%)	
CR+PR	3(6.38%)	5(10.64%)	8(17.02%)	

Abbreviations: AITL, angioimmunoblastic lymphoma; ALCL, anaplastic large cell lymphoma; BM, bone marrow; BV, brentuximab vedotin; CNS, central nerve system; CR, complete remission; CRP, C-reactive protein; CT, chemotherapy; HDACi, histone deacetylase inhibitor; IPI, international prognostic index; LDH, lactate dehydrogenase; MG, microglobulin; N, no treatment; NK/TCL, NK/T cell lymphoma; PD, progressive disease.; PR, partial remission; PTCL, peripheral T-cell lymphoma; RT, radiotherapy; SA, sialic acid; SD, stable disease; TRANS, bone marrow transplantation.

Table S2. P38α ubiquitination site prediction

Position	Code	Peptide	Score
15	K	RPTFYRQELNKTIWEVPERYQ	0.5597
45	K	YGSVCAAFDTKTGLRVAVKKL	0.4527
66	K	SRPFQSIIHAKRTYRELRLLK	0.3429
118	K	LMGADLNNIVKCQKLTDDHVQ	0.2694
152	K	HSADIIHRDLKPSNLAVNEDC	0.7552
165	K	NLAVNEDCELKILDFGLARHT	0.436
248	K	LVGTPGAELLKKISSESARNY	0.3964
249	K	VGTPGAELLKKISSESARNYI	0.481
295	K	LEKMLVLDSDKRITAAQALAH	0.5426
